# The Growth, Pathogenesis, and Secondary Metabolism of *Fusarium verticillioides* Are Epigenetically Modulated by Putative Heterochromatin Protein 1 (FvHP1)

**DOI:** 10.3390/jof11060424

**Published:** 2025-05-31

**Authors:** Andrés G. Jacquat, Natalia S. Podio, María Carmen Cañizares, Pilar A. Velez, Martín G. Theumer, Vanessa A. Areco, María Dolores Garcia-Pedrajas, José S. Dambolena

**Affiliations:** 1 Facultad de Ciencias Exactas Físicas y Naturales (FCEFyN), Universidad Nacional de Córdoba (UNC), Córdoba X5016GCA, Argentina; andresgjacquat@gmail.com; 2 Instituto Multidisciplinario de Biología Vegetal (IMBIV), Consejo Nacional de Investigaciones Científicas y Técnicas (CONICET), Avenida Vélez Sarsfield 1611, Córdoba X5016GCA, Argentina; 3 Instituto de Ciencia y Tecnología de Alimentos Córdoba (ICYTAC), Consejo Nacional de Investigaciones Científicas y Técnicas (CONICET), and Universidad Nacional de Córdoba (UNC). Bv. Dr. Juan Filloy s/n, Cdad. Universitaria, Córdoba X5016BMB, Argentina; npodio@fcq.unc.edu.ar; 4 Instituto de Hortofruticultura Subtropical y Mediterránea “La Mayora”, Universidad de Málaga, Consejo Superior de Investigaciones Científicas (IHSM—UMA—CSIC), Estación Experimental “La Mayora”, Avenida Dr. Wienberg s/n, Algarrobo-Costa, 29750 Málaga, Spain; carmen.canizares@eelm.csic.es; 5 Departamento de Bioquímica Clínica, Facultad de Ciencias Químicas (FCQ), Universidad Nacional de Córdoba (UNC), Córdoba X5000HUA, Argentina; pilarvelez@fcq.unc.edu.ar (P.A.V.); mgtheumer@unc.edu.ar (M.G.T.); 6 Centro de Investigaciones en Bioquímica Clínica e Inmunología (CIBICI), Consejo Nacional de Investigaciones Científicas y Técnicas (CONICET), Haya de la Torre y Medina Allende—Ciudad Universitaria, Córdoba X5000HUA, Argentina; 7 Instituto Multidisciplinario de Investigación y Transferencia Agroalimentaria y Biotecnológica (IMITAB, CONICET-UNVM), Villa María X5900LQC, Argentina; vareco@unvm.edu.ar

**Keywords:** *Fusarium verticillioides*, phytopathogenic, fumonisin B1, fungal secondary metabolite, histone code, epigenetic, heterochromatin, heterochromatin protein 1 (HP1), fusarubin-type naphthoquinones, 8-O-methylnectriafurone

## Abstract

*Fusarium verticillioides* is a globally prevalent phytopathogenic fungus responsible for multiple diseases in maize and a major producer of the mycotoxin fumonisin B1 (FB1), a highly toxic fungal secondary metabolite (FSM). The histone code, which includes reversible modifications such as acetylation and methylation, plays a critical role in regulating chromatin structure and gene expression. In fungi, di- and tri-methylation of histone H3 at lysine 9 (H3K9me2/3) serves as a key epigenetic mark associated with heterochromatin formation and transcriptional repression. In this study, we identified and characterized a putative heterochromatin protein 1 (HP1) family member in *F. verticillioides*, designated FvHP1, based on conserved domain architecture and phylogenetic analyses. FvHP1 retains essential residues required for H3K9me2/3 recognition, supporting its functional conservation within the HP1 protein family. Phenotypic analysis of the ΔFvHP1 mutant revealed impaired vegetative growth, reduced conidiation and virulence, and altered FB1 mycotoxin production. Additionally, the accumulation of red pigment in the mutant was linked to the deregulation of secondary metabolism, specifically the overproduction of fusarubin-type naphthoquinones, such as 8-O-methylnectriafurone. These results support the role of FvHP1 in facultative heterochromatin-mediated repression of sub-telomeric biosynthetic gene clusters, including the pigment-associated PGL1 cluster. Our findings provide new insights into the epigenetic regulation of fungal pathogenicity and metabolite production, as well as the first evidence of a functional HP1 homolog in *F. verticillioides*.

## 1. Introduction

*Fusarium verticillioides* (Sacc.) Nirenberg (teleomorph: *Gibberella fujikuroi* Mating Population A) is a highly prevalent soil-inhabiting filamentous fungus found in the most important cereal crops globally, such as maize (*Zea mays* L., *Poaceae*) [1]. This fungus exhibits both endophytic and pathogenic/parasitic behavior, causing several diseases throughout the maize biological cycle (seedling blight, stalk rot, ear rot) and resulting in crop yield losses [2,3,4,5]. Global corn production losses caused by F. verticillioides are estimated to be 10%, reaching 30% to 50% in severely affected areas. Moreover, it is a major producer of mycotoxins called fumonisins, which pose a significant problem due to their toxicological implications for humans and farm animals [6,7,8,9]. Therefore, given its negative economic impact and its implications for human and animal health, *Fusarium verticillioides* and its secondary metabolites are under continuous study.

The polyketide fumonisin B type 1 (FB1) is the most important fungal secondary metabolite (FSM) produced by *F. verticillioides* due to its high toxicity and prevalence in cereals [8,10]. In addition to FB1, *F. verticillioides* produces other polyketide secondary metabolites such as the naphthoquinone pigment bikaverin [11,12] and black perithecial pigments [11,13]. The compound 8-O-methylnectriafurone was identified as one of several fusarubin-type naphthoquinones produced by Fusarium fujikuroi. These compounds are synthesized through a non-reducing polyketide synthase (NR-PKS) pathway. They are red pigments that contribute to the pigmentation and structural integrity of perithecia, the sexual fruiting bodies of the fungus [13]. Fungal secondary metabolites are intermediates or end products of genus- or species-specific metabolic pathways, whose complete biosynthetic pathways are generally encoded in a biosynthetic gene cluster (BGC) [14,15,16]. This genetic organization is presumed to be the consequence of evolutionary pressure, as it would facilitate both the coordinated transcriptional regulation (co-regulated genes) and the horizontal transfer among strains of all the genetic elements required for the production of a specific secondary metabolite [17,18]. In *F. verticillioides*, BGCs for FB1 and the dark perithecial pigment comprise 15 [19] and 7 genes [11], respectively.

The synthesis of FSMs is tightly regulated by both pathway-specific and broad-domain transcription factors (TFs), as well as by epigenetic mechanisms, including the chemical modification of histones [16,20]. The reversible covalent modifications of histones have an impact on chromatin structure, specifically the facultative heterochromatin region, being an important mechanism to regulate BGCs expression. It is worth mentioning that chromatin presents two main functional and structurally well-defined states: the highly compacted heterochromatin associated with gene silencing, and the unfolded euchromatin associated with gene expression [21]. This transcriptional regulation by the conformational state of chromatin involves the histone code, which refers to the specific pattern of post-translational covalent modifications, such as methylation, acetylation, and phosphorylation, on specific lysine (K), serine (S), and arginine (R) residues of the N-terminus tail of the H3 and H4 histones. Each pattern is linked to a specific biological function, and can be heritable [16,21,22,23]. Specifically, the di-/tri-methylation (me2/3) on lysine 9 of the N-terminal tail of H3 histone (H3-K9me2/3) is among the best-understood post-translational modifications involved in heterochromatin formation and gene repression [24]. The main protein implicated in heterochromatin formation by recognizing and attaching to the H3-K9me2/3 epigenetic mark is heterochromatin protein 1 (HP1) [16,25]. HP1 is a highly conserved eukaryotic heterochromatin-associated non-histone chromosomal protein that contains two major conserved domains: the CHRomatin Organization MOdifier (CHROMO) domain and the CHROMO Shadow domain (CD and CSD, respectively). These domains are separated by a hinge sequence [26,27]. This molecular structure allows HP1 to adopt different spatial conformations, giving it great functional versatility and the capability to interact with many other types of molecules. These include interactions with itself, forming HP1 homodimers, as well as with H3 histone, non-histone chromosomal and non-chromosomal proteins, single and double-stranded DNA, and RNA molecules [28]. Although a wide range of functions have been attributed to HP1 proteins, they are best known for their fundamental role in establishing, propagating, and maintaining the heterochromatic state promoting gene silencing through transcriptional restriction [24,28,29,30,31].

Previously, a lysine histone methyltransferase (KHMTase) encoded by the *FvDim5* (NCBI accession code FVEG_08911) gene was associated with heterochromatin formation in *F. verticillioides* [32]. The biological processes regulated by the expression of the *FvDim5* gene include vegetative growth, microconidia production, pathogenic behavior, and pigmentation (FSM) of the fungal colony [32]. The functional characterization of HP1 in several filamentous fungi has identified it as a major modulator of FSM production, as well as influencing both vegetative and pathogenic behaviors [33,34,35,36]. Nevertheless, no homologous HP1 proteins have been studied in *F. verticillioides* yet. The aim of the present study was to identify and annotate the functional and structural characteristics of the putative HP1 protein encoded by *F. verticillioides*, a reader protein of the epigenetic mark H3K9me2/3, which is catalyzed by the KHMTase FvDim5. Our in silico approach resulted in a unique protein sequence that possesses all the hallmarks of members of the HP1 family. This was considered a putative HP1 protein of *F. verticillioides* and was tentatively named FvHP1 (in non-italics), which is encoded by the *FvHP1* (in italics) gene. Then, our deletion mutant-based approach suggests that FvHP1 plays a significant role in regulating vegetative growth, pathogenicity, and the production of FMSs, such as FB1 and pigments. Although the pathways of FB1 biosynthesis are well-established, this study enhances our understanding of the epigenetic mechanisms that regulate FB1 biosynthesis, as little is known about this aspect. Additionally, we contribute to the structural diversity of fungal pigments by discovering a previously unknown metabolite. Finally, we advance the understanding of the transition to pathogenic and parasitic behavior mediated by epigenetic regulators.

## 2. Materials and Methods

*Fungal strains, growth conditions and conidia production*

*Fusarium verticillioides* M3125 (FGSC #7600), provided by Dr. Robert Proctor (USDA, Agricultural Research Service, National Center for Agricultural Utilization Research, Peoria, IL, USA), served as the wild-type (WT) strain and the parental strain for the deletion mutant used in this study. Fungal strains were grown on Potato Dextrose Agar (PDA) (VWR Chemicals BDH^®^, Leuven, Belgium) or Czapek Dox Agar (CDA) (OXOID, Basingstoke, Hants, UK), in the dark at 25 °C. Czapek Dox Broth (CDB), either commercial (OXOID, Basingstoke, Hants, UK) or homemade (3.0% P/V sucrose, 0.2% NaNO_3_, 0.1% KH_2_PO_4_, 0.04% MgSO_4_ 7H_2_O, 0.05% KCl and 0.0016% Fe(II)SO_4_ 2H_2_O), YEPS (2.0% sacarose, 1.0% yeast extract, 2.0% peptone), and GYAM (0.24 M glucose, 0.05% yeast extract, 8 mM L-asparagine, 5 mM malic, acid, 1.7 mM NaCl, 4.4 mM K_2_HPO_4_, 2 mM MgSO_4_, and 8.8 mM CaCl_2_; pH 3.0) were used for liquid cultures. For the selection of transformants and their subsequent growth, the media were supplemented with 250 µg/mL of Nourseothricin (NTC) (Jena Bioscience, Jena, Deutschland).

*Bioinformatic analyses*

The Basic Local Alignment Search Tool (BLAST) [36] of the NCBI (National Center for Biotechnology Information, U.S. National Library of Medicine, Bethesda, MD, USA) was used for protein sequence searches through the online server at https://blast.ncbi.nlm.nih.gov/Blast.cgi (accessed on 9 March 2024) [37]. The search for domain-level homologies was conducted using the NCBI Conserved Domain Search (CDS) software as implemented at https://www.ncbi.nlm.nih.gov/Structure/cdd/wrpsb.cgi (accessed on 9 March 2024). [38]. Protein alignment was conducted using the probabilistic consistency-based progressive multiple alignment, which incorporates 3D conformational constraints information. This multiple structure-sequence alignment was implemented through the PROMALS3D software (http://prodata.swmed.edu/promals3d/promals3d.php (accessed on 9 March 2024)) [39,40]. For the protein phylogenetic relationships, an unrooted Maximum Likelihood tree of protein sequences was constructed using IQ-TREE multicore version 1.6.11 [41] and displayed with MEGA X version 10.1.5 software [42]. The best-fit substitution model was determined by the ModelFinder program [43]. The alignment was displayed with Unipro UGENE v. 42.0 software [44].

*Production of deletion mutants*

**Figure 1:**
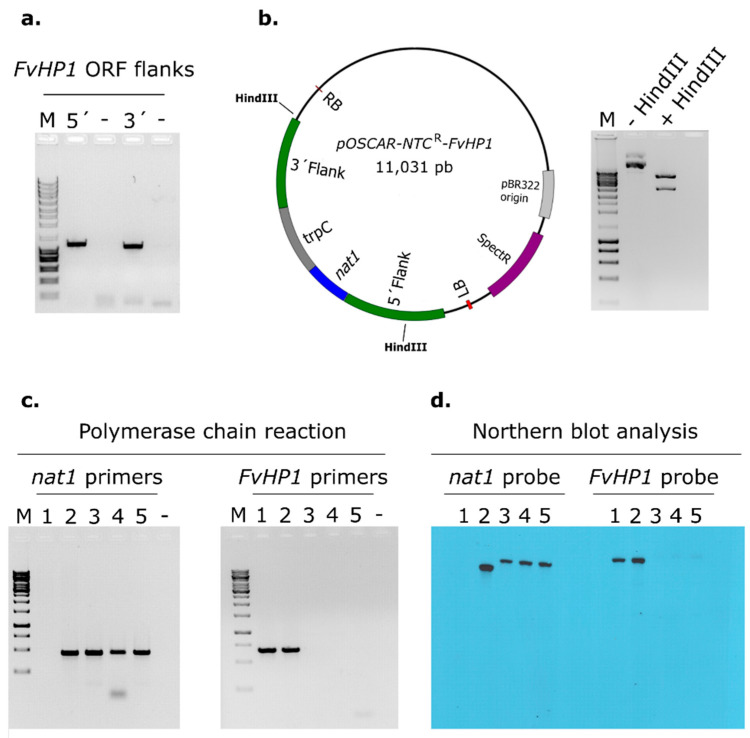
Generation of deletion mutants for *FvHP1* in F. verticillioides. (**a**) Electrophoretic analysis of the *FvHP1* 5′ and 3′ flanks amplified by PCR. (**b**) Map of pOSCAR-NTC^R^-FvHP1 deletion construct (HindIII restriction sites are indicated). The colored boxes indicate functional regions: LB: T-DNA left border; RB: T-DNA right border; nat1: nourseothricin acetyl transferase gene; trpC: promoter region of the Aspergillus nidulans gpdA gene; SpectR: spectinomycin resistance gene; pBR322: replication origin. The electrophoretic gel on the right shows the HindIII digestion pattern of the pOSCAR. (**c**) Confirmation of *FvHP1* deletion by PCR in selected transformants using primer pairs for *FvHP1* gene ORF and nat1 (NTC^R^) gene. (**d**) Southern blot analysis of selected transformants using probes complementary to the *FvHP1* gene ORF and the nat1 gene. Wells labeled with 1, 2, 3, 4, and 5 in panels c and d indicate WT, ECT, and deletion mutant strains ∆HP1#6, ∆HP1#15, and ∆HP1#17, respectively. Wells labeled with “-” in panels (**a**,**c**) indicate PCR negative water control, and in panels (**a**–**c**), well “M“ indicates molecular weight marker (1Kbp, NZYDNA Ladder III, NZYTech^®^, Paço do Lumiar, Lisboa, Portugal)). All electrophoretic analyses were performed on 0.8% *w*/*v* agarose gels stained with ethidium bromide. Bands were revealed under UV light (UVP, LLC, Analytik Jena US LLC, Upland, CA, USA).

For the deletion of FvHP1 in *F. verticillioides,* a deletion vector compatible with *Agrobacterium tumefaciens*-mediated transformation (ATMT) was constructed using the OSCAR method [45]. Approximately 1200 bp of the 5′ and 3′ flanking regions of the gene corresponding to accession code FVEG_01876 were independently amplified by polymerase chain reaction (PCR) using Phusion^®^ High-Fidelity DNA Polymerase (Thermo Fisher Scientific, Waltham, MA, USA) (Figure 1a) [46]. Then, a BP Clonase (BP Clonase^TM^ II, Gateway^TM^, Exime Mix, Invitrogen™, Thermo Fisher Scientific, MA, USA) reaction was performed, combining both amplified gene flanks with the binary vector pOSCAR and a vector providing the resistant marker for the selection of fungal transformants. As resistance marker vector we used pA-NTC^R^-OSCAR, which contains a nourseothricin acetyltransferase (*nat1*) gene conferring resistance to nourseothricin (NTC). The BP clonase reaction was used to transform One Shot™ OmniMAX™ 2 T1R Chemically Competent *E. coli* (Invitrogen, Carlsbad, CA, USA) following the commercial manual’s instructions. The correct construct in *E. coli* colonies was verified by restriction enzyme digestion (Figure 1b). The deletion construct was then transferred to *A. tumafaciens* strain AGL1, using a standard electroporation method, in preparation for fungal transformation. The ATMT of *F. verticillioides* was performed according to previously described protocols [41]. Fungal transformants were confirmed based on their resistance to NTC (250 µg/mL supplemented CDA medium), negative PCR amplification with FvHP1-specific primers, and negative Southern blot hybridization using an FvHP1-specific probe labelled with digoxigenin–dUTP, DNA probe labeling, hybridization, and detection were performed using the DIG High Prime I DNA Labeling and Detection Starter Kit (Roche, Mannheim, Germany). From the total pool of transformant colonies, three were selected and designated as ∆HP1#6, ∆HP1#15, and ∆HP1#17. To confirm stable deletion, transformants were transferred several times to fresh CDA plates supplemented with 250 µg/mL of NTC. Results were displayed in Figure 1, where the PCR product patterns (Figure 1c) and Southern blot analysis (Figure 1d) were consistent with a targeted replacement event in which the ORF of the putative FvHP1 encoding gene (FVEG_0187) was replaced by the expression cassette of the *nat1* gene (NTC resistance-conferring gene). A transformant with a random ectopic integration of the deletion construct was also selected. This strain was designated as ECT. The primers used to amplify the ORF of the FVEG_0187 gene were Hep1F (5′-CCTGCTCTTAGTGACGAGG-3′) and Hep1R (5′-GAGGACTCCAACTCTTCGC-3′). The primers for amplifying the *nat1* gene were NatF (5′-ATGACCACTCTTGACGACACG-3′) and NatR (5′-TTGACGTTGGTGACCTCCAG-3′). To amplify the 5’ flanks of the putative *FvHP1* gene ORF (1329 bp), the primers used were FvHP1-attB2r (5′-ggggacagctttcttgtacaaagtggaaCAGCCAACGACATCTCATCTCTG-3′) and FvHP1-attB1r (5′-ggggactgcttttttgtacaaacttgtACTAACGTGTGCTAGTCTACGTTG-3′). For amplifying the 3’ flanks of the putative *FvHP1* gene ORF (1187 bp), the primers were FvHP1-attB4 (5′-ggggacaactttgtatagaaaagttgttCGAGACGCAGCCATATAAGGT-3′) and FvHP1-attB3 (5′-ggggacaactttgtataataaagttgtAGAGACGAGCTTCCAGATACC-3′). Lowercase indicates the sequence attached to the end of each primer for recognition by BP clonase.

*Lag phase, growth rate, and conidiation*

To assess fungal vegetative growth, PDA Petri dishes were inoculated at the center with 5 μL of a conidia suspension (1.0 × 10^6^ conidia/mL) from each strain and incubated in the dark at 25 ± 1 °C until the colonies completely covered the surface of the plate. For each colony, two diameter measurements were recorded daily using a Vernier scale caliper. The two diameter measurements were taken perpendicular to each other, and an average was recorded. The lag phase was estimated using a nonlinear regression curve derived from a non-modified Gompertz model [47,48] fitted to the colony growth over an 8-day incubation period. This vegetative colonial feature represents the time (in hours) it takes for the colony to reach an area of 13.4 mm^2^ (equivalent to a diameter of 5 mm). Mathematically, the lag phase was determined by solving for the time (“t”) variable when “A” = 13.4 (Equation (1)). The growth rate was estimated from the slope of the linear regression curve fitted to data derived from the linear vegetative growth phase. To determine microconidia production, 5 mL of YEPS liquid medium was inoculated with 10 µL of a conidia suspension (1.0 × 10^6^ conidia/mL) in 50 mL glass tubes. The tubes were placed on an orbital shaker in the dark at 28 ± 1 °C for four days. Then, the culture was filtered through Miracloth membranes, and the microconidia were counted using a hemocytometer.

A = α × e ^[−β × e^(−γ × t)]^ (1)

where “A” is the area variable (mm^2^), “t” is the time variable (hours), “e” is the mathematical constant ~2.718, and “α”, “β” and “γ” are the statistics of the Gompertz equation. In all assays, “α”, “β” and “γ” significantly explained the observed variables at a significance level of 0.05.

*Pathogenicity assays*

Fungus–maize interaction assays were conducted under two different culture regimes: hydroponic–phytotron and soil–greenhouse. They were designated as experimental schemes A and B, respectively. In addition to these, an in vitro assay was performed to evaluate radicle development. In all experiments, kernels were disinfected with a NaClO solution (3.0 mg Cl/mL) and subjected to thermal treatment at 60 °C for 5 min [49]. For experimental scheme A, maize seeds (hybrid ACA 474 VT3Pro MG-RR2, Argentina) were soaked in a conidia suspension or sterile water (mock inoculation) for 10 min. Then, the grains were placed on Petri dishes containing 2% water agar (WA) medium and incubated in darkness at 25 ± 1 °C for three days. Upon germination, seeds were transferred to hydroponic culture with a 12/12 h photoperiod, a temperature of 25 ± 1 °C, and a relative humidity of 75 ± 10%. The irrigation regime and the concentrations of the macro and micronutrients in the hydroponic solution were set according to the method described by Zörb et al. [50]. Stem heights were measured at 6, 10, and 14 days post-infection (DPI). The experiment was conducted once with 10–12 plants per treatment. For experimental scheme B, seeds (hybrid Golden Bantam sweet corn, Rocalba S.A., Girona, Spain) were incubated in sterile distilled water overnight in darkness at 25 ± 1 °C to induce germination. Then, they were individually planted in 1 L plastic pots containing a mixture of non-autoclaved substrate, coconut fiber, and vermiculite. The pots were then placed in the greenhouse, where they were exposed to natural photoperiod, relative humidity ranging from 60% to 95%, and temperatures ranging from 15 °C to 28 °C. At 10 days post-planting (DPP), seedlings were infected with a suspension of 1.0 × 10^6^conidia/mL injected into the stem. At this point (10 DPP), the height of the seedlings (h0) was measured. At 10 days post-inoculation (DPI) or 20 DPP, seedling height (h1) was measured again to calculate the “post-infection seedling relative growth” parameter as a general plant health estimator. The equation implemented was (h1 − h0) × (h0)^−1^ and the result was expressed as a percentage. Three replicates of the assay were performed, with 7–14 seedlings per treatment in each replicate.

Radicle growth was used as another general plant health estimator. For this assay, 12 to 15 corn seeds (ACA 474 VT3Pro MG-RR2, Pergamino, Buenos Aires, Argentina) for each treatment were immersed in a 1.0 × 10^6^conidia/mL suspension in PBS buffer with 1.5% Tween-20 for 10 min. A 1.5% Tween-20 in PBS buffer without conidia was used as the negative control. Then, they were placed in glass Petri dishes (90 × 15 mm) containing 1.5% WA medium and kept in darkness at 25 ± 1 °C for three days. They were symmetrically arranged around the periphery of the dish, with the kernel tips facing towards the center of the dish. The entire experiment was carried out twice.

*FB1 production*

To investigate the impact of the gene deletion on FB1 production, two different culture media were employed: GYAM and CDB. In *F. verticillioides* WT strains, FB1 synthesis is induced in GYAM medium but not in CDB medium. Liquid cultures were prepared by inoculating 50 mL of GYAM or CDB with 0.5 mL of conidia suspension (1.0 × 10^6^ conidia/mL). After seven days of incubation in an orbital shaker at 200 rpm at a temperature of 28 ± 1 °C in the dark, a 1 mL sample was taken from each culture and centrifuged at 9000 RCF for 15 min to remove fungal cells. The supernatants were transferred to a fresh tube and diluted with HPLC-grade acetonitrile (Sintorgan, Villa Martelli, Buenos Aires, Argentina) at a 1:1 ratio. The FB1 content was determined using high-performance liquid chromatography (HPLC) (PerkinElmer Inc., Waltham, MA, USA) coupled with a fluorescence detector [51]. The quantification of FB1 was carried out by comparing the peak areas with those obtained from samples containing FB1 analytical standards (PROMEC Unit, South African Medical Research Council, Francie van Zijl Drive, Tygerberg Hospital, Parow, Tygerberg 7505, Cape Town, South Africa). The experiment was repeated twice, and five independent cultures were analyzed for each strain.

*Production, isolation, and identification of fungal metabolites*

Fungal metabolites were extracted and purified from a 7-day CDB culture (200 RPM orbital shaking in dark at 25 ± 1 °C) after removing fungal cells by centrifugation and filtration (0.45 PVDF filters). Solid-phase extraction C-18 cartridges (500 mg, 6 mL, 40 μm) conditioned with 10 mL of methanol and 10 mL of water were used. Filtrates were eluted from the columns using 2 mL of HPLC-grade acetonitrile and stored at −70 °C for further characterization. The metabolites were analyzed by chromatography coupled to mass spectrometry (HPLC-MS) using an Agilent Technologies 1200 Series UHPLC (Agilent, Santa Clara, CA, USA) equipped with a gradient pump (Agilent G1312B SL Binary), solvent degasser (Agilent G1379B), and an autosampler (Agilent G1367D SL þ WP). The chromatographic separation was achieved on a C18 column (5 mm, 100 mm × 4.60 mm i.d.) (Agilent, Santa Clara, CA, USA) at 40 °C using an Agilent G1316B column heater module. The mobile phase consisted of a solution of 5 mM ammonium acetate and 0.4% formic acid (solvent A) and a solution of 5 mM ammonium acetate and 0.4% formic acid in methanol (solvent B). The solvent gradient started with 5% B for 1 min and then changed to 50% B over 3 min. This was followed by a second ramp to 65% B over 6 min and a third ramp to 98% B over 12 min. The solvent remained in this state for 7 min. Then, an 8 min washing and stabilization step was used before the next run. The flow rate was set at 0.3 mL/min, and the injection volume was 10 µL. The HPLC system was connected to a photodiode array detector (PAD SL, Agilent G1315C) in conjunction with an ESI source and Q-TOF mass spectrometer (micrOTOF-QII Series, Bruker Daltonics Inc., Billerica, MA, USA). UV-vis spectra were recorded from 200 to 700 nm. Mass spectra were recorded in positive ion mode from *m*/*z* 80 to 1200. The working conditions for the ionization source were as follows: a capillary voltage of 4500 V, a nebulizer gas pressure of 4.0 bar, a drying gas flow of 8.0 L/min, and a drying gas temperature of 200 °C. Nitrogen was used as the nebulizer/dryer gas, while argon was used as the collision gas. The instrument was operated in full-scan mode. The exact mass was verified by introducing a sodium formate solution (40 mM) at the end of each chromatographic run using the multipath valve of the Micro-QTOF II. Metabolite identification was based on an accurate *m*/*z* ratio (mass error ≤ 5 ppm) and fragmentation pattern (MS/MS spectrum with collision energies of 10.0, 15.0, and 20.0 eV). Data acquisition and spectrum processing were performed using Compass 3.1 and DataAnalysis 4.1, respectively (BrukerDaltonics, Billerica, MA, USA). Molecular features (pairs *m*/*z*; retention time) were extracted from the raw spectra using ProfileAnalysis 2.3 software (BrukerDaltonics, MA, USA). LC-MS analyses were processed using the Find Molecular Features (FMF) function with S/N = 10 for peak detection. Background subtraction and time alignment were also performed. Advanced bucket generation was used, with a retention time range of 1–30 min, a mass range of 80–1200 *m*/*z*, and each bucket (spectral bin) was formed with a resolution of 0.3 min and 10 *m*/*z* mDa without normalization. The generated bucket table was exported to Excel, and a blank subtraction step was performed. The final feature matrix was normalized by summing the peak areas for univariate analysis (volcano plot, *p* < 0.05 and Fold Change ≥ 2) and Pareto scaled for multivariate supervised statistical modeling (OPLS-DA) using MetaboAnalyst 5.0 online software.

*Statistical analyses*

Statistical analyses of fungal growth (*Lag phase, growth rate, and conidiation*), fumonisin B1 production data and phytopathogenicity assay data were performed by one-way analysis of variance (ANOVA) (*p* ≤ 0.05). The normality and homogeneity of variance were tested. Data are presented as mean ± standard error and differences between means were considered significant if probability (*p*) ≤ 0.05. DGC test was used for means comparisons. All statistical analyses were performed using InfoStat v2020 software.

## 3. Results

### 3.1. Identification of a Putative HP1 Protein in Fusarium verticillioides

**Figure 2:**
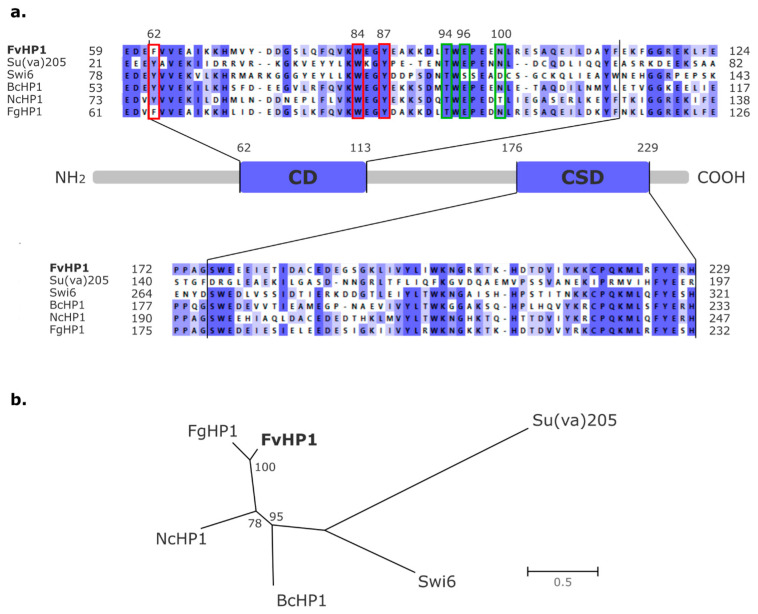
FvHP1 Functional-structural annotation, sequence comparison, and phylogenetic relationship. (**a**) A box diagram representing the complete amino acid sequence of the FvHP1 protein shows the CD and CSD, indicated by amino acid residue positions, along with the respective segmented PROMALS3D alignment. Color intensity indicates the percentage of identity for each site. Key sites for identifying the H3K9me2/3 histone mark are highlighted in red (aromatic) and green (non-aromatic) frames, with their corresponding amino acid residue position indicated. (**b**) Unrooted maximum likelihood phylogenetic tree of the complete aa sequence of orthologous HP1 proteins. Alignment: PROMALS3D. Best-fit amino acid substitution model: VT + F + G4. Scale bar: average number of substitutions per site.

We scanned the NCBI Protein Reference Sequences (NCBI RefSeq_protein) database for putative HP1 proteins using a *F. verticillioides*-limited (taxid:117187) BLASTp search. The HP1 protein from *F. graminearum* (NCBI accession number XP_011319962.1) served as the query. The best hit showed 100% query coverage, 70.52% identity, and an e-value of 3.0 × 10^−116^ with the protein sequence recorded under NCBI accession code: XP_018744913.1. Two other hits displayed 31% and 32% query coverage, 35.53% and 37.04% identity, and e-values of 2.0 × 10^−10^ and 3.0 × 10^−05^, respectively. We proceeded with the analysis of the top BLASTp result, due to its highly favorable characteristics, indicating it as the most likely homologous protein, as detailed below. The hypothetical protein XP_018744913.1 consists of 246 amino acid (aa) residues. NCBI’s Conserved Domain Search (CDS) tools indicated the presence of two evolutionarily conserved domains among HP1 family members: the amino-terminal chromo domain (CD) and the carboxyl-terminal chromo shadow domain (CSD) [26]. The CD spans positions 62 to 113 aa, while the CSD spans positions 176 to 229 aa. A multiple structure-sequence alignment (Figure 2a), which includes several previously characterized HP1 proteins, demonstrated that the CD and CSD of the *F. verticillioides* XP_018744913.1 protein exhibit a high degree of similarity to the well-defined CD and CSD of Su(var)205 isoform A (NCBI accession code: NP_476755.1) [29,52,53], Swi6 (NP_593449.1) [54], BcHP1 (NCBI accession code: ATZ56699.1; Old locus tag: BC1G_06432) [55], NcHP1 [56], and FgHP1 [35] proteins. Furthermore, the candidate *F. verticillioides* HP1 (XP_018744913.1) was found to contain three aromatic residues (phenylalanine 62, tryptophan 84, and tyrosine 87) and three non-aromatic residues (threonine 94, glutamate 96, and asparagine 100) at homologous sites. These residues have been determined to be key for recognizing the H3K9me2/3 mark [57] (see Supplementary Figure S1). The protein phylogenetic reconstruction showed that XP_018744913.1 (from *F. verticillioides*) and FgHP1 (from *F. graminearum*) share a recent common ancestor (Figure 2b). In summary, the similarities of the CD and CSD regions and the evolutionary relationships with other well-characterized members of the HP1 protein family provide sufficient evidence to propose that the amino acid sequence recorded by *F. verticillioides* under the NCBI accession code XP_018744913.1 is homologous to HP1 proteins, hereafter referred to as FvHP1 (non-italic letters). The coding nucleotide sequence, recorded under the NCBI accession code FVEG_01876, will be hereafter referred to as *FvHP1* (italic letters).

### 3.2. Deletion of FvHP1 Gene Affects the Growth, Asexual Reproduction, and Pigment Production

**Figure 3:**
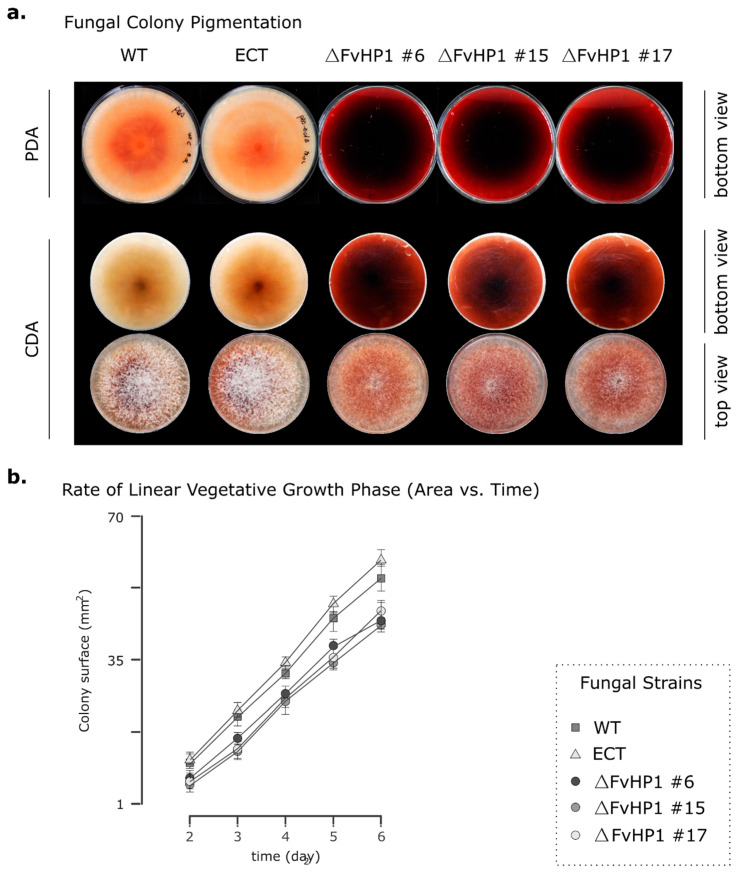
Effect of *FvHP1* deletion on colony pigmentation and vegetative growth. (**a**) Colony pigmentation. Photographs show bottom and top views of colonies. Strains were grown in CDA (OXOID™) and PDA (VWR Chemicals BDH^®^) media for 35 days in darkness at 25 °C. (**b**) Vegetative growth. Dots represent the mean area (mm^2^) of *F. verticillioides* WT and mutant colonies during their linear growth state at different incubation times, from 2 to 6 days. Whiskers indicate the standard deviation. Strains were incubated on PDA in the dark at 25 ± 1 °C. Values having different letters are significantly different from each treatment according to DGC test of multiple range (*p* ≤ 0.05). Experiments were performed twice with 5 replicates for each strain.

To analyze the plausible functions of the *F. verticillioides* HP1 protein, we obtained three independent stable NTC-resistant transformants. These were single-spore sub-cultured (monosporic culture) and designated as ∆*FvHP1* #6, ∆*FvHP1* #15, and ∆*FvHP1* #17 strains (Figure 1c). An NTC-resistant transformant with a random ectopic integration of the deletion construct, referred to as the ECT strain, was included, in our experiments, as a control alongside the parental WT strain. This helped to rule out any potential effects of the *nat1* gene product or other alterations induced during the transformation process on the fungus phenotype. Interestingly, in contrast to the light-colored colonies of the WT and ECT strains, the three ∆*FvHP1* strains exhibited dark, deep red pigmentation when grown on solid media (Figure 3a). This strong pigment accumulation was also observed and measured in liquid CDB medium, as described later. These observations indicate that the FvHP1 protein participates in pigment production in *F. verticillioides*.

All three deletion strains exhibited a lower rate of vegetative growth compared to the WT and ECT strains (Figure 3b), suggesting that the FvHP1 protein may play a key role in vegetative behavior. For a comprehensive evaluation of the effect of *FvHP1* deletion on the colony’s vegetative characteristics such as lag phase, growth rate, and conidiation, we selected one of the mutant strains, the *F. verticillioides* ∆*FvHP1* #6, for all subsequent evaluations. The lag phase of the deletion mutant was significantly longer (38 h; 95% CI 30–41) compared to that of the WT (23 h; 95% CI 15–27) and ECT (24 h; 95% CI 19–27) strains (Table 1). Besides, the growth rate of the ∆*FvHP1* #6 strain was significantly slower than that of the WT and ECT strains, with the mutation affecting the growth rate by approximately 15.9% and 11.4%, respectively. On the other hand, no significant difference in growth rate was found between the WT and ECT strains (Table 1). As expected, the extended lag phase and reduced growth rate resulted in significant differences in colony size. This difference was clearly observed at 5 days post-inoculation, showing a significant reduction in growth area compared to WT and ECT strains (25.7% and 31.7%, respectively). Additionally, the mutation had a negative effect on the production of microconidia (Table 1). Δ*FvHP1* #6 produced significantly fewer conidia than the WT (66.7%) and ECT (55.5%) strains under conditions that stimulate conidia production. Taken together, these results indicate that FvHP1 protein plays an important role in regulating the vegetative growth of *F. verticillioides.*

### 3.3. FvHP1 Protein Is Required for Normal Fumonisin B1 Production

**Figure 4:**
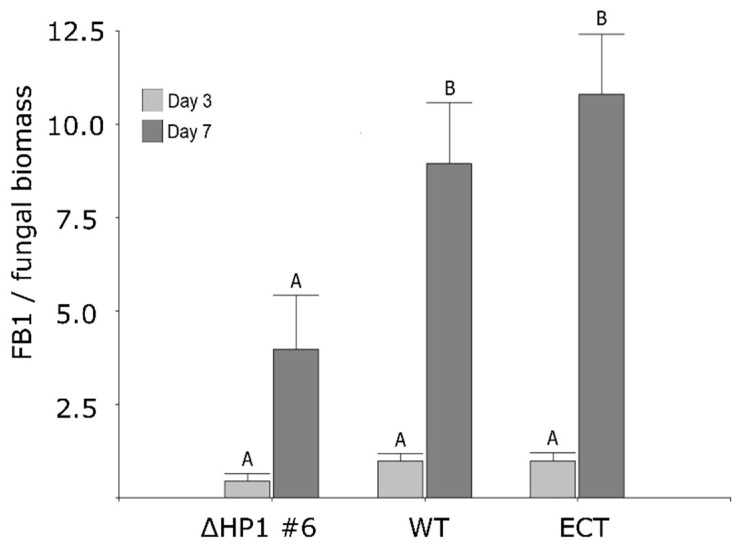
Effect of FvHP1 deletion on fumonisin B1production. Values are expressed as means (µg of FB1/mg of mycelium biomass), and whiskers represent the standard error. Bars with different letters are statistically different from each other according to DGC test of multiple range (*p* ≤ 0.05), for the same incubation time (3 and 7 days).

The effect of deleting the *FvHP1* gene on the production of fumonisin B1 (FB1) was also assessed using two different media: GYAM medium, which induces FB1synthesis, and CDB, which does not induce the synthesis of this mycotoxin. CDB medium was used to determine if *FvHP1* deletion would result in FB1 production under non-inducing conditions. At 3 days post-inoculation, all three fungal strains evaluated exhibited comparable levels of FB1 production in GYAM medium (Figure 4). However, at 7 days post-inoculation, the amount of FB1 produced by the deleted strain was significantly lower than that produced by the control strains: 3.97 ± 1.45 µg/mg compared to 8.95 ± 1.62 µg/mg (WT) and 10.80 ± 1.62 µg/mg (ECT). These results indicate a positive role for FvHP1 protein in regulating FB1 production. On the other hand, none of the evaluated strains showed FB1 production in CDB medium.

### 3.4. FvHP1 Plays a Role in the Pathogenicity of F. verticillioides

**Figure 5:**
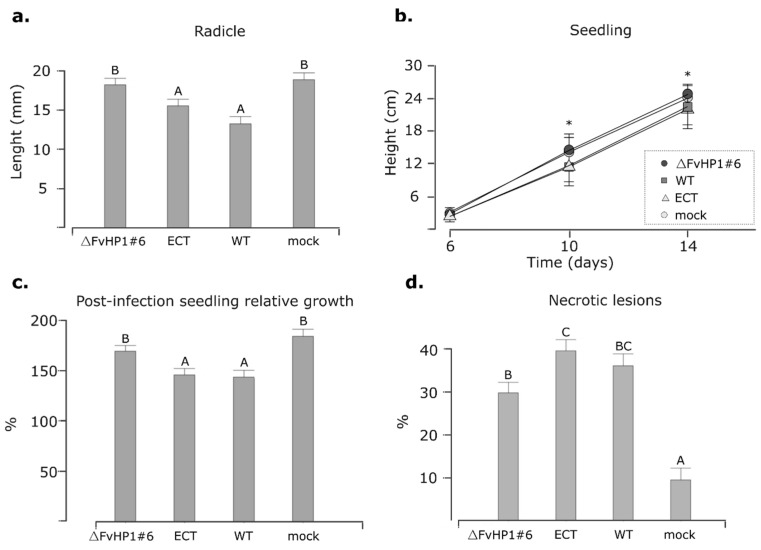
Effect of *FvHP1* gene deletion on F. verticillioides pathogenicity towards maize. (**a**) Radicle development assay. Corn seeds were surface-infected and incubated in a Petri dish with WA medium for 3 days, after which the radicle length was recorded. Values are expressed as mean with standard deviation (whiskers). Two experimental replicates were conducted, with 12–15 seeds per treatment in each replicate. (**b**) Hydroponic-phytotron assay (experimental schemes A): Height measurements are expressed as the mean, with whiskers indicating the standard deviation. Asterisk indicates significant differences between treatments (DMRT; α = 0.05). The experiment was conducted once with 10–12 plants per treatment. (**c**,**d**) Greenhouse pathogenicity assay (experimental schemes B). These panels represent data from three experimental replicates and 7–14 seedlings per treatment in each replicate. The “post-infection seedling relative growth” parameter was calculated as the percentage increase in stem length at 10 DPI relative to the initial stem length (10 DPP). The “Necrotic lesions” parameter, defined as leaves with at least one coalescing and/or localized necrotic region, was assessed at 10 DPI and quantified as the percentage of leaf area with lesions per seedling (Supplementary Figure S2). In panels a, c, d, different letters among treatments indicate statistical differences according to DGC test of multiple range (*p* ≤ 0.05).

When present in the soil or crop residues, *F. verticillioides* hyphae can colonize maize root tissues and the mesocotyl within a few days of seed germination [3]. Moreover, they can directly penetrate the grain’s pericarp shortly after sowing [3,58]. Given this, we estimated the virulence of *F. verticillioides* by measuring radicle growth rate and stem growth rate under well-controlled laboratory conditions. The radicle growth rate (Figure 5a) and stem growth rate (Figure 5b) were used here as general indicators of plant health. For these experiments, maize seeds were infected by immersing them in conidia suspensions. Subsequently, one group was designated for radicle length measurements, while another group was transferred to hydroponic irrigation in a growth chamber. Additionally, we assessed the pathogenicity of the deletion mutants under other maize infection and cultivation conditions. This involved inducing an infection by directly injecting a conidial suspension into the stem of a 10-day post-planting (DPP) seedling grown in a pot. At 20 DPP, we evaluated the stem growth from the day of infection (10 days post-infection: 10 DPI) (Figure 5c) and assessed the extent of necrotic lesions on the leaves (Figure 5d).

The infection with the WT strain resulted in statistically significant differences in radicle growth rate, seedling growth rate in the hydroponic–phytotron assay, post-infection seedling relative growth in soil-greenhouse assays and necrotic lesions when compared to the non-infected plants (mock treatment). Consequently, these quantitative parameters were utilized to estimate the degree of pathogenicity of the mutant strains, with the WT and mock treatment serving as reference values. In panels “a”, “b” and “c” of Figure 5, the results showed that deletion mutant Δ*FvHP1*#6 acquired values that resembled the mock treatment with respect to the WT and ECT infection. The similar results observed between infections with the WT strain and the ECT strain suggest that the random ectopic integration of the *nat1* gene expression cassette did not significantly modify the pathogenic behavior of the parental strain. Analyzing each experiment specifically, the Δ*FvHP1*#6 strain did not generate any significant effect on radicle growth compared to the seeds treated with sterile water. In the phytotron–hydroponic plant culture system, where seeds were superficially infected with conidia (Figure 5b), the growth of seedlings infected with the ECT and WT strains did not differ statistically from each other, but it was lower than that of seedlings infected with the deletion strain and non-infected seedlings. The differences became significantly more pronounced at 10 and 14 days post-infection (DPI). Similarly, in the greenhouse plant culture system (Figure 5, panels c and d), where the infection was generated by injecting conidia directly into the stem, a similar result was observed. The post-infection seedling relative growth generated by the deleted strain was statistically equivalent to that of non-infected plants. In the analysis of necrotic lesions, the fungal disease signature was not completely abolished in the Δ*FvHP1* #6 strain. The visible damage induced by infection with this strain was significantly higher than that observed in the uninfected control seedlings with respect to the percentage of leaves with necrotic lesions (Figure 5d). Necrotic lesions in seedlings infected with the Δ*FvHP1* #6 strain were not significantly smaller than those observed in seedlings infected with WT and ECT strains. The results of the pathogenicity assays suggest that the putative FvHP1 protein is associated with the pathogenic development of *F. verticillioides* in maize; however, its involvement is not entirely determinative of virulent behavior.

### 3.5. FvHP1 Is a Negative Regulator of Naphthoquinone Pigment Synthesis in F. verticillioides

**Figure 6:**
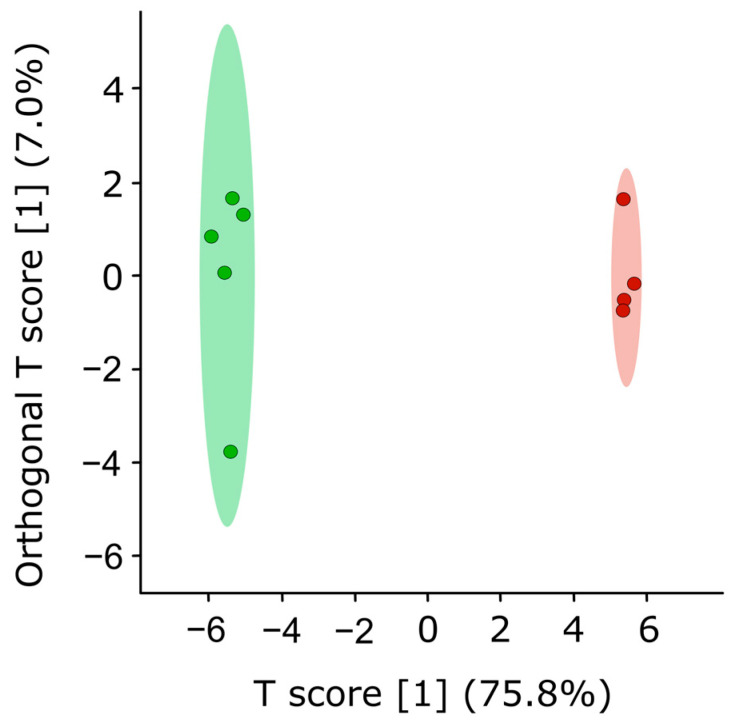
OPLS-DA Scores plot. This Orthogonal Partial Least Squares Discriminant Analysis (OPLS-DA) scores plot discriminates between the metabolite profiles of fungal strains. Samples were normalized by sum and Pareto scaled. Green and red dots indicate samples from ECT and Δ*FvHP1* #6 strains, respectively.

The pigmentation observed in cultures of the deleted strain was analyzed. Out of 44 total metabolites present in both the Δ*FvHP1* #6 strain and ECT strain culture medium (CDB), 29 exhibited significant differences in concentration. A multivariate OPLS-DA analysis was conducted to identify the most important metabolites differentiating the profiles of the two strains. The scores plot (Figure 6) shows clear discrimination between the samples being evaluated. The validation parameters demonstrated that a satisfactory model was obtained, with R2Y and Q2 values of 0.987 and 0.983, respectively (Figure S3B). R2Y and Q2 parameters greater than 0.5 and close to 1 reveal the strong correlation (R2Y) and predictive power (Q2) of the model for discriminating fungal samples [59]. To ensure that the difference explained by the OPLS-DA model was the result of a real effect caused by the defining factor, rather than a random effect, a permutation test using 2000 permutations was performed (Figure S3C). The test demonstrated that the model was not statistically different from any of the permuted models (*p* < 0.05), indicating no overfitting of the data. In addition, the areas under the curve (AUC) of the receiver operating characteristic (ROC) curve were used to evaluate the overall predictive power of the OPLS-DA models (Figure S3D). Significantly high AUC values (AUC = 1) were obtained, validating the predictive power of the OPLS-DA.

According to the OPLS-DA analysis, twenty metabolites showed a VIP value greater than 1 (Figure S3E). Then, an S-Plot analysis was used to identify metabolites that were statistically significant and potentially biologically relevant, considering their contributions to the model and their reliability. In total, six metabolites were highlighted in the S-Plot (Figure S3F). Finally, both univariate (Volcano Plot) and multivariate (VIP and S-Plot) metrics were considered to identify the most significant metabolites for discriminating between extracts of ∆*FvHP1* #6 and ECT strains. A total of 5 metabolites were selected: V3, V8, V16, V24, and V29. Four of them were produced at significantly higher levels in the deleted mutant compared to the ECT strain, while one metabolite showed decreased production in the deletion mutant (Figure S4). The selected compounds were tentatively identified based on their exact mass, isotope patterns, mass fragmentation patterns, UV–Vis spectra, and information from literature and online databases (Table 2).

Metabolite V3 showed a precursor ion at *m*/*z* 421.1085 [M + H]+. This compound was assigned to level IV of identification [60] as it is an unknown metabolite not previously reported in the literature. Its UV spectrum (Figure S5) showed absorption maximums at 227 and 271 nm, and its isotopic profile indicated the presence of a Zn atom. The molecular formula of this compound is C_20_H_24_N_2_O_4_Zn. The MS/MS spectrum showed a first fragment at *m*/*z* 377.12 [M+ H − 44]+ corresponding to the loss of CO_2_. The next fragment at *m*/*z* 351.13 [M + H − 70]+ indicated the loss of CO_2_ + C_2_H_2_.

Metabolite V8 was assigned to level II of identification (Figure S6). This compound showed a UV-Vis spectrum with maximums at 253, 318, and, 444 nm, and a precursor ion at *m*/*z* 319.0797 [M + H]+. Its isotopic profile and mass exact allowed identifying its molecular formula (C_16_H_16_O_7_). The MS/MS spectrum shows a first fragment at *m*/*z* 301.07 [M + H − 18]+ (loss of H_2_O) and a second fragment at *m*/*z* 286.04 [M + H − 33]+ (loss of H_2_O + CH_3_•). Besides, fragments at *m*/*z* 304.05 ([M + H − CH_3_•]+), 289.03 ([M + H − CH_3_• − CH_3_•]+) and 272.06 ([M + H − H_2_ − HO• − CO]+) were also registered. A similar metabolite was reported by Studt et al. [13] in *F. fujikuroi*. Thus, this metabolite was tentatively identified as 8-O-Methylnectriafurone.

Metabolites V16 and V29 showed a similar precursor ion at *m*/*z* 301.070 ([M + H]+) and a similar UV-Vis spectrum (Figures S7 and S8). Their isotopic profiles and mass exact indicated a molecular formula of C_16_H_12_O_6_. These metabolites also showed a similar fragmentation pattern, with fragments at *m*/*z* 288.060 ([M + 3H − CH_3_•]+), 273.037 ([M + H − C_2_H_4_]+), 257.043 ([M + H − C_2_H_4_ − C_2_H_4_]+), 245.041 ([M + H − C_2_H_4_ − CO]+), 243.061 ([M + 3H − CH_3_CO_2_H]+), and 229.047 ([M + 3H − CH_3_CO_2_H − CH_2_]+). These fragments were also observed in the MS2 spectrum (*m*/*z* 301.070) of the metabolite V8 (Figure S6), suggesting that metabolites V16 and V29 could be isomers of this fragment. Both V16 and V29 were assigned to level III of identification, belonging to the naphthoquinones family.

Finally, metabolite V24 showed a precursor ion at *m*/*z* 436.1012 [M + H]+. This compound was assigned to level IV of identification. Its UV spectrum (Figure S9) showed maximum absorption at 223 nm, and its isotopic profile and mass exact indicated the molecular formula C_23_H_17_NO_8_. MS/MS spectrum showed a first fragment at *m*/*z* 421.075 [M + H − CH_3_•] + and a second fragment at 408.101 [M + H − CO] +. Fragments at *m*/*z* 393.080 ([M + H − CH_3_• − CO] +) and 375.070 ([M + H − CH_3_• − CO − H_2_O]+) were also observed. In total, five metabolites (V3, V8, V16, V24, and V29) are reported for the first time for *F. verticillioides* in this work. Three of them are naphthoquinone pigments produced at high levels by the ∆*FvHP1* #6 strain.

## 4. Discussion

**Figure 7:**
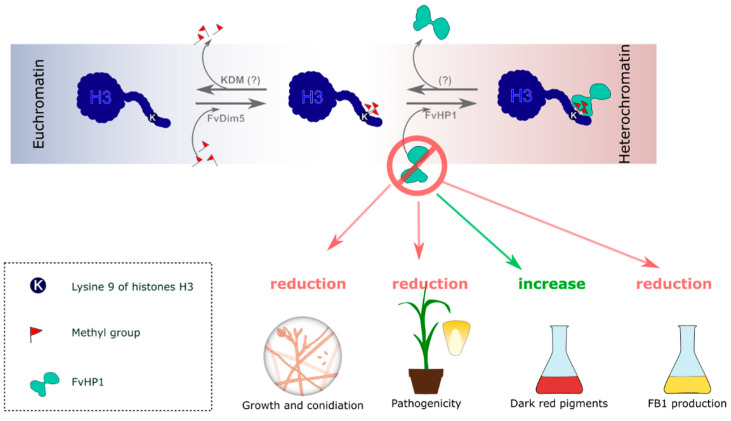
Schematic representation of the role of putative FvHP1 in heterochromatin formation and the biological effects of its absence. In the euchromatin state, the di- and tri-methylation of the deacetylated K9 of histone H3 is catalyzed by the specific methyltransferase FvDim5. This epigenetic mark is recognized by the putative FvHP1 protein, which contributes to chromatin compaction. The deletion of *FvHP1* negatively affects vegetative growth, conidiation, virulence, and FB1 production, while simultaneously increasing the accumulation of dark red pigments.

The histone code, which involves the reversible acetylation, methylation, ubiquitination, and phosphorylation patterns on the N-terminal tails and globular domains of histones H2B, H3, and H4, influences the dynamics between heterochromatin and euchromatin, consequently affecting various cellular functions [22]. In fungi, methylation and acetylation patterns on lysine 9 of the N-terminal tail of H3 histone (H3K9) are one of the most extensively studied epigenetic marks within the histone code [16]. A histone methyltransferase (HMT) catalyzes the specific di-/tri-methylation (me2/3) of H3K9. This specific H3K9 me2/3 modification binds to an HP1 protein, resulting in a non-covalent H3K9me2/3-HP1 interaction. This event triggers a cascade of changes in the conformation of the heterochromatin state [24,25]. In this context, an H3K9 HMT, named FvDim5, was characterized in *F. verticillioides* [32]. However, to the best of our knowledge, no putative HP1 protein has been characterized in this species until now. In this study, we identified the protein sequence registered under the NCBI-RefSeq accession number XP_018744916.1, encoded by the annotated gene FVEG_01876, as a putative HP1 protein in *F. verticillioides*. We refer to this as the FvHP1 protein (Figure 7), and our phylogenetic evidence suggests it is an orthologous protein to the HP1 of *F. graminearum*. The aa sequence of FvHP1 shows a high similarity with the two typical domains shared by all members of the HP1 family: the CD and CSD protein motifs. Moreover, their aa sequence contains the conserved sites most significant for recognizing the H3K9me2/3 histone epigenetic mark, as reported by Nielsen et al. [57]. That is, the FvHP1 CD domain has three aa residues in homologous sites to *Mus musculus* (Mm) HP1b protein (Y21, W42, and F45), which are necessary to maintain the hydrophobic pocket structure for recognizing H3K9me2/3. The W residue at position 84 aa of FvHP1 is conserved with respect to the homologous MmHP1b W42 site, a site that does not permit point substitution to preserve CD functionality [57]. However, two point mutations were recorded at the other two essential sites for CD functionality. Specifically, the FvHP1 protein has two amino acid substitutions at the homologous Y21 and F45 sites of MmHP1b: Y62F and F87Y mutations in FvHP1 (see Supplemental Figure S1). These substitutions, Y by F and vice versa (both amino acid residues with a hydrophobic aromatic ring), presumably do not significantly affect CD domain activity as they do not alter the properties of the hydrophobic pocket, according to Nielsen et al. [57]. Thus, Y62F and F87Y are evolutionarily admissible point substitutions. Moreover, three other side-chain amino acids essential for interacting with H3K9me2/3, namely T51, E53, and N57 in MmHP1b protein [57], are also found at homologous sites on the FvHP1 protein (T94, E96, and N100, respectively; see Supplemental Figure S1). On the other hand, our phylogenetic reconstruction indicated that the predicted FvHP1 protein shares a recent common ancestor with FgHP1 protein, the HP1 family member encoded by *F. graminearum*. This result is consistent with the close evolutionary relationship of these fungal species [61,62]. Our analyses, based on sequence similarity and phylogenetic reconstruction, strongly suggest that FvHP1 is a putative protein belonging to the HP1 family and is orthologous to the HP1 protein of *F. graminearum*. This report provides the first characterization of a member of the HP1 family in *F. verticillioides*.

The sequence analysis performed in the present study shows that FvHP1 retains the aa residues necessary to ensure CD functionality, at least for recognizing histones epigenetic marks. However, its specific role in *F. verticillioides* metabolism needs to be elucidated. While a basal function can be attributed to all members of the protein family, HP1 appears to have a species-specific role that depends on the molecular environment at the chromosomal level [24,28,63], a phenomenon reflected across different fungal species. We found that deleting the *FvHP1* gene impairs the vegetative growth of the fungus and its clonal reproduction, specifically the production of microconidia. This is in agreement with what was reported by Freitag et al. [56], who demonstrated a similar role for HP1 in the vegetative growth of *Neurospora crassa*. Furthermore, our results are consistent with those obtained by Gu et al. [32], which showed that the KHMTase FvDim5, responsible for methylating the K9 residues in histone 3 (allowing the FvHP1 protein to recognize the epigenetic mark), plays a role in normal vegetative growth and microconidia production in *F. verticillioides* [32]. In contrast, only a slight reduction in radial growth was observed when the HP1 encoded gene was deleted in *Epichloë festucae* [64], and no defect in vegetative growth was discernible in HP1 deletion mutants in *F. graminearun* [35], *Aspergillus nidulans* [34], and *Botrytis cinerea* [55]. The reduced vegetative behavior was accompanied by a decrease in the fungal virulence of *F. verticillioides* in its natural host. All quantitative infection signs evaluated in our study (root and stem growth, and leaf lesions) indicated that pathogenic behavior appears to be impaired with the deletion of the *FvHP1* gene. In agreement with this result, it was determined that the normal expression of the *FvDim5* gene is necessary to maintain the pathogenic behavior of *F. verticillioides* [32]. Our experimental approaches allowed us to evaluate the natural colonization capability of fungi on maize tissues. The radicle length assay and stem growth rate under phytotron-hydroponic conditions were conducted to simulate fungal infection by allowing microconidia to adhere to the kernel surface. The germination of the conidia likely occurred outside the plant tissues, with hyphae colonizing interstitial and cellular spaces and breaking physical plant barriers to initiate a systemic infection. In the greenhouse assay, we focused on the infection caused by the massive germination of conidia within the plant tissues. Here, infection was facilitated because the hyphae did not have to penetrate the cuticles and plant epidermises. Both infection strategies resulted in significantly reduced pathogenic behavior. This likely stems from the impaired vegetative growth and reduced microconidia production. The ability of *F. verticillioides* to behave as an endophyte or pathogen/parasite depends on its growth mode in the host, which in turn depends on the genotype of both the specimens and the environment [3,58,65].

Mycotoxins are regarded as species-specific FSM and their role in pathogenic behavior has been previously established in *F. verticillioides* through the production of fumonisins, with FB1 being a key factor involved in its pathogenicity [66,67,68,69]. We investigated the effect of deleting the *FvHP1* gene on FB1 production. As expected, the deleted strain showed no FB1 production under non-FB1-inductive conditions (CDB medium), but reduced production was observed under FB1-inductive conditions (GYAM medium). Visentin et al. [70] revealed that histone acetylation acts as a positive regulator of the *FUM1* and *FUM21* genes by utilizing a standard histone deacetylase inhibitor (trichostatin A, TSA) in the fungus cultured on CDB medium. It is pertinent to remember that a hyperacetylated state of chromatin is related to its euchromatin form (non-compact and transcriptionally active chromatin). However, no increase in FB1 production was recorded since the *FUM8* gene, which encodes an aminotransferase required to form the biologically active FB1, was downregulated under TSA treatment [70]. The non-production of FB1 measured in CDB medium in both experimental studies (ours and 65) suggests that FB1 BGC is under another regulatory mechanism and the chromatin state is of secondary relevance in this specific non-inducing condition. Other regulatory mechanisms could be involved, such as the broad-domain transcription factor PAC1, which is implicated in the repression of FB1 synthesis under alkaline conditions like CDB medium [71]. On the other hand, the results obtained under FB1-inducing medium demonstrated that the deletion of FvHP1 had a negative effect on FB1 production. This effect was unexpected because the presumed chromatin state in our deleted fungus would be lax and transcriptionally active. This result, however, is in line with the decrease in virulence observed in mutant *F. verticillioides*. Corn tissues create a conducive environment for hyphal growth and promote the production of fumonisins, similar to the GYAM medium (in vitro method). Therefore, despite the lack of quantification of FB1 in plant tissue (no assay performed), it is known that FB1 is produced by *F. verticillioides* whether it behaves as a parasite or endophyte [58].

In contrast to our findings, Gu et al. [32] demonstrated that the deletion of *FvDim5* had a positive effect on FB1 production in maize kernels. However, this discrepancy could be due to the fact that FB1 evaluation was performed under different environmental conditions (GYAM medium and maize kernels). To assess the impact of *FvHP1* deletion on the chromatin conformation and methylation level near the promoters of genes in the FB1 BGC, a chromatin immunoprecipitation assay should be conducted. Nevertheless, we preliminarily demonstrate that the production of FB1 is partially regulated by FvHP1, which aligns with the previously mentioned decrease in virulence. To fully understand the regulation of FB1 biosynthesis, it is essential to conduct an in-depth study of the interactions between specific and broad-domain transcription factors, as well as the acetylation and methylation patterns of the K9 residue on the H3 protein. Ultimately, our results achieve the goal of phenotypically characterizing the absence of the *FvHP1* gene and assessing the functionality of the FvHP1 protein at the organismal level in *F. verticillioides*, particularly concerning FB1 biosynthesis.

The intense red pigmentation observed in Δ*FvHP1* strains suggested that the synthesis of certain secondary metabolites in *F. verticillioides* might be regulated by conformational/condensation state of the chromatin. It is necessary to emphasize that the BGC responsible for producing these deep red pigments appears to be primarily regulated by the chromatin conformation, unlike the cluster responsible for synthesizing FB1. The role of reversible histone modifications affecting chromatin structure in the regulation of the synthesis of various FSMs, such as fungal pigments, has been demonstrated in other fungal species [35]. To date, two different types of pigments have been reported in *F. verticillioides*: bikaverin and the uncharacterized fusarubin-type naphthoquinones associated with perithecium dark pigmentation [11,13] Here, we delved deeper into elucidating the chemical nature of these red pigments. In total, we found that three naphthoquinone-like pigments were highly accumulated in *F. verticillioides* Δ*FvHP1* grown in CDB medium. On the other hand, bikaverin was not detected under these growth conditions. This is not surprising because CDB is an alkaline medium, and bikaverin biosynthesis is known to be repressed in alkaline conditions by the regulatory factor PAC1 [72,73]. Analysis of the accurate *m*/*z* ratio (mass error ≤ 5 ppm), fragmentation pattern, and absorption spectra of the three metabolites enabled us to identify 8-O-methylnectriafurone as one of the pigments highly accumulated in the Δ*FvHP1* strain. Although the other two naphthoquinone-like metabolites that highly accumulated in the *FvHP1* gene deletion mutant could not be fully identified, their accurate *m*/*z* ratio and fragmentation patterns were similar to that of 8-O-methylnectriafurone. These results suggest that they are structurally related to 8-O-methylnectriafurone. Prior to the present report, the production of 8-O-methylnectriafurone by *F. verticillioides* had not been documented, to the best of our knowledge. However, this metabolite was previously found to be a component of pigments in the perithecium of *F. fujikuroi* [13]. In this work, we provide the first evidence regarding the chemical structure of the perithecium dark pigments in *F. verticillioides*.

The perithecium pigments of *F. fujikuroi* and *F. graminearum* are different. They belong to the chemical family of fusarubins and bostrycoidins, respectively. The similarity between the chemical structures of the perithecium pigments found in this study and those reported for *F. fujikuroi* suggests that the perithecium pigments in *F. verticillioides* are fusarubins and originate from the seven-member gene cluster named PGL1. Bioinformatic studies using genomic data could further support this hypothesis. Sequence alignment by Frandsen et al. [74] showed that the putative PGL1 BGC of *F. verticillioides* exhibited high similarity to those of *F. fujikuroi* and *F. graminearum*. Specifically, a synteny region exists in the PGL1 BGCs of *F. verticillioides* and *F. fujikuroi,* where the six homologous core genes of the cluster conserve both their relative positions and orientations in the two species [74]. One of these core genes, *PGL1*, encodes a seven-domain non-reducing polyketide synthase (NR-PKS) highly conserved in *F. verticillioides* and *F. fujikuroi* [13]. These NR-PKSs are predicted to form a fusarubin-type naphthoquinone structure by condensing seven acetyl-CoA units, which constitutes the chemical backbone of 8-*O*-methylnectriafurone [11,13]. On the other hand, in *F. graminearum*, there is an extra gene in its PGL1 BGC, the *PglL* gene, which is absent from the homologous region in the *F. fujikuroi* and *F. verticillioides* PGL BGCs [74]. Consequently, in the case of *F. graminearum,* the intermediate metabolite produced by the NR-PKS of the PGL BGC, 6-O-demethylfusarubinaldehyde, undergoes different secondary chemical modifications, leading to the formation of bostrycoidins instead of fusarubin structures.

Finally, although the chemical structure of the perithecium pigments of *F. verticillioides* has not been completely determined, our identification of 8-O-methylnectriafurone and related chemical compounds, combined with evidence from bioinformatic studies using genomic data from evolutionarily related species, provides sufficient evidence to propose their origin in the fusarubin chemical pathway coded by the PGL1 BGC. This pathway appears to be regulated by the FvHP1-mediated conformational state of the chromatin. The PGL1 BGC of *F. verticillioides* is located sub-telomerically, meaning it is expected to be transcriptionally repressed by facultative heterochromatin during the vegetative state. The identification of these pigments as highly accumulated in the Δ*FvHP1* strain provides evidence to support the hypothesis that in *F. verticillioides,* the PGL1 BGC is repressed during vegetative growth through the formation of facultative heterochromatin mediated by FvHP1 activity. In agreement with our results, a previous study reported by Reyes-Dominguez et al. [35] demonstrated the involvement of FgHP1 in the negative regulation of aurofusarin biosynthesis by *F. graminearum.*

Given that in other fungal species these pigments, fusarubin-type naphthoquinones, including 8-O-methylnectriafurone, have been associated with perithecium pigmentation [13,74] and the protection of ascospores (inside the perithecium) from ultraviolet light and the reactive oxygen species [75], the involvement of FvHP1 in regulating these fusarubins metabolites found in our work suggests that changes in chromatin conformation could play a role in regulating the sexual state in *F. verticillioides* (or *Gibberella fujikuroi* MP A, its teleomorph state). This hypothesis aligns with the findings of Cam et al. [14], who discovered that proteins homologous to HP1 are strongly associated with mating in *Schizosaccharomyces pombe*. Additionally, this corresponds with the observation that these pigments not only color the perithecia but are also believed to play a role in maintaining the structural integrity of perithecia in *F. fujikuroi* [13]. Furthermore, this is in agreement with the findings reported by Gu et al. [32], who showed that the *FvDim5* deletion mutant exhibited a lack of perithecial formation in *Gibberella fujikuroi* MP A. To our knowledge, the finding of 8-O-methylnectriafurone red pigment production by *F. verticillioides* reported in the present paper represents the first characterization of perithecial pigments in this fungal species. Although it is not directly shown to be part of perithecia, there is sufficient evidence to strongly support this hypothesis. Experiments on the role of HP1 in perithecia pigmentation are currently being conducted.

In several organisms, including fungi, certain epigenetic marks are inherited during mitotic and meiotic cell division, allowing specific gene expression states to remain stable across successive cell generations [76,77,78,79,80]. This phenomenon is not implausible and merits further investigation, particularly in the context of chromatin compaction states in filamentous fungi.

## Figures and Tables

**Table 1:** Effect of *FvHP1* gene deletion on fungal vegetative features.

Strain	Lag Phase (95% CI) ^1^	Growth Rate ^2^	Colony Size at 5 Days of Incubation (mm^2^) ^3^	Conidiation (Conidia/mL) ^4^
∆*FvHP1* #6	38 (30–41)	412 ± 7 a	1841 ± 110 a	4 ± 2 × 10^7^ a
ECT	24 (19–27)	466 ± 7 b	2695 ± 104 b	9 ± 1 × 10^7^ b
WT	23 (15–27)	490 ± 9 b	2477 ± 104 b	12 ± 1 ×10^7^ c

^1^ Time (hours) with a 95% confidence interval (CI) (lower limit—upper limit). Time was estimated from a nonlinear regression fitted to the Gompertz model (see “Materials and Methods”) using colony size (area) data from 0 to 8 days of incubation (in darkness at 25 ± 1 °C) after point inoculation, at the center of a 90 × 15 Petri dish, with PDA (Britania S.A., Ciudad Autónoma de Buenos Aires, Argentina, Buenos Aires, Argentina) medium. Five cultures were made for each strain. ^2^ The growth rate was calculated based on the slope (mm^2^/day ± standard error) derived from a linear regression during the linear growth phase. This phase encompassed the incubation period (in darkness at 25 ± 1 °C) from 2 to 6 days after point inoculation, at the center of a 90 × 15 Petri dish, with PDA (Britania S.A.) medium. ^3^ Colony size was recorded at the end of a 5-day incubation period (in darkness at 25 ± 1 °C) after point inoculation, at the center of a 90 × 15 Petri dish, with PDA (Britania S.A.) medium. ^4^ Conidia production was determined in YEPS liquid medium. Values having different letters are significantly different from each treatment according to DGC test of multiple range (*p* ≤ 0.05). Data obtained from 5 independent fungal cultures.

**Table 2:** Metabolite identification.

Metabolite	RT ^1^ (min)	Experimental ^2^ [M + H]+ *m*/*z*	Calculated ^3^ [M + H]+ *m*/*z*	Tentative M. Formula	Δm ^4^ (ppm)	# Sigma ^5^	MS Fragmentation (Intensity) ^6^	MS2 Fragmentation (Intensity) ^7^	l (nm) ^8^	Putative Compound	MSI ^9^
V3	8.18	421.1085	421.1071	C20H24N2O4Zn	3.7	5.5	421 (16); 377 (25); 351 (100); 260 (20); 242 (6)		224; 271		IV
V8	9.80	319.0797	319.0812	C16H14O7	4.7	4.6	319 (42); 304 (41); 301 (100); 289 (36); 286 (81); 273 (35); 272 (53); 261 (19); 258 (25); 257 (35); 249 (14); 243 (40); 229 (10)	301 → 303 (52); 289 (18); 288 (70); 273 (100); 270 (13); 261 (48); 260 (26); 259 (41); 245 (56); 246 (33); 243 (7); 233 (37); 229 (11) 303 → 303 (62); 289 (15); 288 (80); 273 (100); 261 (38); 260 (35); 259 (61); 246 (25); 245 (42); 233 (46)	224; 258sh; 304; 444	8-O-Methylnectriafurone	II
V16	11.08	301.0699	301.0707	C16H12O6	2.7	66.4	303 (7); 301 (5); 289 (14); 288 (100); 287 (17); 273 (72); 257 (11); 245 (23); 243 (9); 229 (6)	288 -> 289 (35); 288 (13); 274 (89); 271 (51); 256 (33); 247 (93); 246 (91); 245 (32); 244 (44); 243 (100); 233 (34); 231 (66); 229 (70)	268; 344; 506	Naphthoquinone	III
V24	12.00	436.1012	436.1013	C23H17NO8	3.6	2.6	436 (100); 421 (49); 420 (33); 408 (12); 403 (10); 393 (39); 379 (13); 375 (48); 358 (6); 347 (11); 330 (11); 304 (19); 286 (41); 258 (8)		223		IV
V29	13.57	301.0705	301.0707	C16H12O6	0.4	16.3	301 (22); 303 (10); 288 (100); 286 (23); 273 (71); 272 (40); 258 (36); 257 (93); 245 (21); 243 (96); 242 (32); 229 (45)		265; 344; 514	Naphthoquinone	III

^1^ RT: retention time, ^2^ Experimental [M + H]+ m/: Experimental mass of the protonated molecular ion, ^3^ Calculated [M + H]+ *m*/*z*: Calculated mass of the protonated molecular ion, ^4^ Δm: Mass deviation (ppm), difference between the experimental mass and the theoretical mass, expressed in parts per million (ppm). ^5^ # Sigma: It represents the deviation of the experimental mass from the theoretical mass in terms of standard deviations (σ). ^6^ MS fragmentation (intensity): Relationship between the ion fragments generated during analysis and the intensity of their signals in the mass spectrum. Energy of fragmentation equal to 15 eV except metabolite V24 (20 eV). ^7^ MS2 fragmentation (intensity): Ion fragments generated in the second stage of tandem mass spectrometry (MS/MS or MS^2^). ^8^ l (nm): Wavelength of maximum absorption (nm). ^9^ MSI: Mass Spectrometry Imaging identification level.

## Data Availability

The original contributions presented in this study are included in the article/Supplementary Material. Further inquiries can be directed to the corresponding authors.

## Supplementary Materials

The following supporting information can be downloaded at: https://www.mdpi.com/article/10.3390/jof11060424/s1, Figure S1: Chromo domain sequences pair alignment (NCBI MSA Viewer v1.20.1) of FvHP1 (this study) and mouse HP1β (also known as MOD1 or M31). Figure S2: Signs of necrosis on a maize seedling leaf. Figure S3: Orthogonal Partial Least Squares Discriminant Analysis (OPLS-DA) to discriminate metabolite fungal strain. Figure S4: Box Plot of the most important metabolites to discriminate between ∆FvHP1 and FvECT strains. Figure S5: UV-Vis, MS, and MSMS spectrums of Metabolite V3. Figure S6: UV-Vis, MS and MSMS spectrums of Metabolite V8. MS 2 spectrums were also included. Figure S7: UV-Vis, MS, and MSMS spectrums of Metabolite V16. Figure S8: UV-Vis, MS, and MSMS spectrums of Metabolite V24. Figure S9: UV-Vis, MS, and MSMS spectrums of Metabolite V29.

## References

[1] Blacutt A.A., Gold S.E., Voss K.A., Gao M., Glenn A.E. (2018). *Fusarium verticillioides*: Advancements in Understanding the Toxicity, Virulence, and Niche Adaptations of a Model Mycotoxigenic Pathogen of Maize. Phytopathology.

[2] Bacon C.W., Hinton D.M. (1996). Symptomless Endophytic Colonization of Maize by *Fusarium moniliforme*. Can. J. Bot..

[3] Oren L., Ezrati S., Cohen D., Sharon A. (2003). Early Events in the *Fusarium verticillioides*—Maize Interaction Characterized by Using a Green Fluorescent Protein-Expressing Transgenic Isolate. Appl. Environ. Microbiol..

[4] Oldenburg E., Höppner F., Ellner F., Weinert J. (2017). Fusarium Diseases of Maize Associated with Mycotoxin Contamination of Agricultural Products Intended to Be Used for Food and Feed. Mycotoxin Res..

[5] Gai X., Dong H., Wang S., Liu B., Zhang Z., Li X., Gao Z. (2018). Infection Cycle of Maize Stalk Rot and Ear Rot Caused by *Fusarium verticillioides*. PLoS ONE.

[6] Glenn A.E. (2007). Mycotoxigenic *Fusarium* Species in Animal Feed. Anim. Feed Sci. Technol..

[7] Presello D.A., Botta G., Iglesias J., Eyhérabide G.H. (2008). Effect of Disease Severity on Yield and Grain Fumonisin Concentration of Maize Hybrids Inoculated with *Fusarium verticillioides*. Crop Prot..

[8] Braun M.S., Wink M. (2018). Exposure, Occurrence, and Chemistry of Fumonisins and Their Cryptic Derivatives. Comp. Rev. Food Sci. Food Safe.

[9] Kamle M., Mahato D.K., Devi S., Lee K.E., Kang S.G., Kumar P. (2019). Fumonisins: Impact on Agriculture, Food, and Human Health and Their Management Strategies. Toxins.

[10] Nelson P.E., Plattner R.D., Shackelford D.D., Desjardins A.E. (1991). Production of Fumonisins by *Fusarium moniliforme* Strains from Various Substrates and Geographic Areas. Appl. Environ. Microbiol..

[11] Brown D.W., Butchko R.A.E., Busman M., Proctor R.H. (2012). Identification of Gene Clusters Associated with Fusaric Acid, Fusarin, and Perithecial Pigment Production in *Fusarium verticillioides*. Fungal Genet. Biol..

[12] Achimón F., Krapacher C.R., Jacquat A.G., Pizzolitto R.P., Zygadlo J.A. (2021). Carbon Sources to Enhance the Biosynthesis of Useful Secondary Metabolites in *Fusarium verticillioides* Submerged Cultures. World J. Microbiol. Biotechnol..

[13] Studt L., Wiemann P., Kleigrewe K., Humpf H.-U., Tudzynski B. (2012). Biosynthesis of Fusarubins Accounts for Pigmentation of *Fusarium fujikuroi* Perithecia. Appl. Environ. Microbiol..

[14] Cam H.P., Sugiyama T., Chen E.S., Chen X., FitzGerald P.C., Grewal S.I.S. (2005). Comprehensive Analysis of Heterochromatin- and RNAi-Mediated Epigenetic Control of the Fission Yeast Genome. Nat. Genet..

[15] Palmer J.M., Keller N.P. (2010). Secondary Metabolism in Fungi: Does Chromosomal Location Matter?. Curr. Opin. Microbiol..

[16] Strauss J., Reyes-Dominguez Y. (2011). Regulation of Secondary Metabolism by Chromatin Structure and Epigenetic Codes. Fungal Genet. Biol..

[17] Khaldi N., Collemare J., Lebrun M.-H., Wolfe K.H. (2008). Evidence for Horizontal Transfer of a Secondary Metabolite Gene Cluster between Fungi. Genome Biol..

[18] Tralamazza S.M., Rocha L.O., Oggenfuss U., Corrêa B., Croll D. (2019). Complex Evolutionary Origins of Specialized Metabolite Gene Cluster Diversity among the Plant Pathogenic Fungi of the *Fusarium graminearum* Species Complex. Genome Biol. Evol..

[19] Proctor R.H., Brown D.W., Plattner R.D., Desjardins A.E. (2003). Co-Expression of 15 Contiguous Genes Delineates a Fumonisin Biosynthetic Gene Cluster in Gibberella Moniliformis. Fungal Genet. Biol..

[20] Yu J.-H., Keller N. (2005). Regulation of Secondary Metabolism in Filamentous Fungi. Annu. Rev. Phytopathol..

[21] Grewal S.I.S., Jia S. (2007). Heterochromatin Revisited. Nat. Rev. Genet..

[22] Strahl B.D., Allis C.D. (2000). The Language of Covalent Histone Modifications. Nature.

[23] O’Kane C.J., Hyland E.M. (2019). Yeast Epigenetics: The Inheritance of Histone Modification States. Biosci. Rep..

[24] Eissenberg J.C., Elgin S.C.R. (2014). HP1a: A Structural Chromosomal Protein Regulating Transcription. Trends Genet..

[25] Machida S., Takizawa Y., Ishimaru M., Sugita Y., Sekine S., Nakayama J., Wolf M., Kurumizaka H. (2018). Structural Basis of Heterochromatin Formation by Human HP1. Mol. Cell.

[26] Eissenberg J.C., Elgin S.C. (2000). The HP1 Protein Family: Getting a Grip on Chromatin. Curr. Opin. Genet. Dev..

[27] Lomberk G., Wallrath L., Urrutia R. (2006). The Heterochromatin Protein 1 family. Genome Biol..

[28] Kumar A., Kono H. (2020). Heterochromatin Protein 1 (HP1): Interactions with Itself and Chromatin Components. Biophys. Rev..

[29] Eissenberg J.C., Morris G.D., Reuter G., Hartnett T. (1992). The Heterochromatin-Associated Protein HP-1 Is an Essential Protein in Drosophila with Dosage-Dependent Effects on Position-Effect Variegation. Genetics.

[30] Fanti L., Berloco M., Piacentini L., Pimpinelli S. (2003). Chromosomal Distribution of Heterochromatin Protein 1 (HP1) in *Drosophila*: A Cytological Map of Euchromatic HP1 Binding Sites. Genetica.

[31] Tortora M.M.C., Brennan L.D., Karpen G., Jost D. (2023). HP1-Driven Phase Separation Recapitulates the Thermodynamics and Kinetics of Heterochromatin Condensate Formation. Proc. Natl. Acad. Sci. USA.

[32] Gu Q., Ji T., Sun X., Huang H., Zhang H., Lu X., Wu L., Huo R., Wu H., Gao X. (2017). Histone H3 Lysine 9 Methyltransferase FvDim5 Regulates Fungal Development, Pathogenicity and Osmotic Stress Responses in *Fusarium verticillioides*. FEMS Microbiol. Lett..

[33] Bok J.W., Noordermeer D., Kale S.P., Keller N.P. (2006). Secondary Metabolic Gene Cluster Silencing in *Aspergillus nidulans*. Mol. Microbiol..

[34] Reyes-Dominguez Y., Bok J.W., Berger H., Shwab E.K., Basheer A., Gallmetzer A., Scazzocchio C., Keller N., Strauss J. (2010). Heterochromatic Marks Are Associated with the Repression of Secondary Metabolism Clusters in *Aspergillus nidulans*: Heterochromatin Regulation of Secondary Metabolism. Mol. Microbiol..

[35] Reyes-Dominguez Y., Boedi S., Sulyok M., Wiesenberger G., Stoppacher N., Krska R., Strauss J. (2012). Heterochromatin Influences the Secondary Metabolite Profile in the Plant Pathogen *Fusarium graminearum*. Fungal Genet. Biol..

[36] Nishitani A., Hiramatsu K., Kadooka C., Mori K., Okutsu K., Yoshizaki Y., Takamine K., Tashiro K., Goto M., Tamaki H. (2023). Expression of Heterochromatin Protein 1 Affects Citric Acid Production in *Aspergillus luchuensis* Mut. kawachii. J. Biosci. Bioeng..

[37] Altschul S.F., Gish W., Miller W., Myers E.W., Lipman D.J. (1990). Basic Local Alignment Search Tool. J. Mol. Biol..

[38] Marchler-Bauer A., Bryant S.H. (2004). CD-Search: Protein Domain Annotations on the Fly. Nucleic Acids Res..

[39] Pei J., Grishin N.V. (2007). PROMALS: Towards Accurate Multiple Sequence Alignments of Distantly Related Proteins. Bioinformatics.

[40] Pei J., Kim B.-H., Grishin N.V. (2008). PROMALS3D: A Tool for Multiple Protein Sequence and Structure Alignments. Nucleic Acids Res..

[41] Nguyen L.-T., Schmidt H.A., Von Haeseler A., Minh B.Q. (2015). IQ-TREE: A Fast and Effective Stochastic Algorithm for Estimating Maximum-Likelihood Phylogenies. Mol. Biol. Evol..

[42] Kumar S., Stecher G., Li M., Knyaz C., Tamura K. (2018). MEGA X: Molecular Evolutionary Genetics Analysis across Computing Platforms. Mol. Biol. Evol..

[43] Kalyaanamoorthy S., Minh B.Q., Wong T.K.F., Von Haeseler A., Jermiin L.S. (2017). ModelFinder: Fast Model Selection for Accurate Phylogenetic Estimates. Nat. Methods.

[44] Okonechnikov K., Golosova O., Fursov M., the UGENE team (2012). Unipro UGENE: A Unified Bioinformatics Toolkit. Bioinformatics.

[45] Paz Z., García-Pedrajas M.D., Andrews D.L., Klosterman S.J., Baeza-Montañez L., Gold S.E. (2011). One Step Construction of Agrobacterium-Recombination-Ready-Plasmids (OSCAR), an Efficient and Robust Tool for ATMT Based Gene Deletion Construction in Fungi. Fungal Genet. Biol..

[46] Mullins E.D., Chen X., Romaine P., Raina R., Geiser D.M., Kang S. (2001). *Agrobacterium*-Mediated Transformation of *Fusarium oxysporum*: An Efficient Tool for Insertional Mutagenesis and Gene Transfer. Phytopathology.

[47] Ochoa-Velasco C.E., Navarro-Cruz A.R., Vera-López O., Palou E., Avila-Sosa R. (2018). Growth Modeling to Control (in Vitro) *Fusarium verticillioides* and *Rhizopus stolonifer* with Thymol and Carvacrol. Rev. Argent. Microbiol..

[48] Di Rienzo J.A., Casanoves F., Balzarini M.G., Gonzalez L., Tablada M., Robledo C.W. (2020). InfoStat Versión 2020. Centro de Transferencia InfoStat, FCA, Universidad Nacional de Córdoba, Argentina. http://www.infostat.com.ar.

[49] Bacon C.W. (1994). A Corn Seedling Assay for Resistance to *Fusarium moniliforme*. Plant Dis..

[50] Zörb C., Geilfus C.-M., Mühling K.H., Ludwig-Müller J. (2013). The Influence of Salt Stress on ABA and Auxin Concentrations in Two Maize Cultivars Differing in Salt Resistance. J. Plant Physiol..

[51] Shephard G.S., Sydenham E.W., Thiel P.G., Gelderblom W.C.A. (1990). Quantitative determination of fumonisins B1 and B2 by high-performance liquid chromatography with fluorescence detection. J. Liq. Chromatogr..

[52] Eissenberg J.C., James T.C., Foster-Hartnett D.M., Hartnett T., Ngan V., Elgin S.C. (1990). Mutation in a Heterochromatin-Specific Chromosomal Protein Is Associated with Suppression of Position-Effect Variegation in Drosophila Melanogaster. Proc. Natl. Acad. Sci. USA.

[53] Elgin S.C.R., Reuter G. (2013). Position-Effect Variegation, Heterochromatin Formation, and Gene Silencing in Drosophila. Cold Spring Harb. Perspect. Biol..

[54] Lorentz A., Ostermann K., Fleck O., Schmidt H. (1994). Switching Gene Swi6, Involved in Repression of Silent Mating-Type Loci in Fission Yeast, Encodes a Homologue of Chromatin-Associated Proteins from Drosophila and Mammals. Gene.

[55] Zhang X., Liu X., Zhao Y., Cheng J., Xie J., Fu Y., Jiang D., Chen T. (2016). Histone H3 Lysine 9 Methyltransferase DIM5 Is Required for the Development and Virulence of *Botrytis cinerea*. Front. Microbiol..

[56] Freitag M., Hickey P.C., Khlafallah T.K., Read N.D., Selker E.U. (2004). HP1 Is Essential for DNA Methylation in Neurospora. Mol. Cell.

[57] Nielsen P.R., Nietlispach D., Mott H.R., Callaghan J., Bannister A., Kouzarides T., Murzin A.G., Murzina N.V., Laue E.D. (2002). Structure of the HP1 Chromodomain Bound to Histone H3 Methylated at Lysine 9. Nature.

[58] Bacon C.W., Yates I.E., Hinton D.M., Meredith F. (2001). Biological Control of *Fusarium moniliforme* in Maize. Environ. Health Perspect..

[59] Ramos M., Ghosson H., Raviglione D., Bertrand C., Salvia M.-V. (2022). Untargeted Metabolomics as a Tool to Monitor Biocontrol Product Residues’ Fate on Field-Treated Prunus Persica. Sci. Total Environ..

[60] Sumner L.W., Amberg A., Barrett D., Beale M.H., Beger R., Daykin C.A., Fan T.W.-M., Fiehn O., Goodacre R., Griffin J.L. (2007). Proposed Minimum Reporting Standards for Chemical Analysis: Chemical Analysis Working Group (CAWG) Metabolomics Standards Initiative (MSI). Metabolomics.

[61] Ma L.-J., van der Does H.C., Borkovich K.A., Coleman J.J., Daboussi M.-J., Di Pietro A., Dufresne M., Freitag M., Grabherr M., Henrissat B. (2010). Comparative Genomics Reveals Mobile Pathogenicity Chromosomes in *Fusarium*. Nature.

[62] Watanabe M., Yonezawa T., Lee K., Kumagai S., Sugita-Konishi Y., Goto K., Hara-Kudo Y. (2011). Molecular Phylogeny of the Higher and Lower Taxonomy of the *Fusarium*genus and Differences in the Evolutionary Histories of Multiple Genes. BMC Evol. Biol..

[63] Casale A.M., Cappucci U., Piacentini L. (2021). Unravelling HP1 Functions: Post-Transcriptional Regulation of Stem Cell Fate. Chromosoma.

[64] Chujo T., Lukito Y., Eaton C.J., Dupont P.-Y., Johnson L.J., Winter D., Cox M.P., Scott B. (2019). Complex Epigenetic Regulation of Alkaloid Biosynthesis and Host Interaction by Heterochromatin Protein I in a Fungal Endophyte-Plant Symbiosis. Fungal Genet. Biol..

[65] Lanubile A., Ferrarini A., Maschietto V., Delledonne M., Marocco A., Bellin D. (2014). Functional Genomic Analysis of Constitutive and Inducible Defense Responses to *Fusarium verticillioides* Infection in Maize Genotypes with Contrasting Ear Rot Resistance. BMC Genom..

[66] Glenn A.E., Zitomer N.C., Zimeri A.M., Williams L.D., Riley R.T., Proctor R.H. (2008). Transformation-Mediated Complementation of a *FUM* Gene Cluster Deletion in *Fusarium verticillioides* Restores Both Fumonisin Production and Pathogenicity on Maize Seedlings. Mol. Plant-Microbe Interact..

[67] Brown D.W., Busman M., Proctor R.H. (2014). *Fusarium verticillioides SGE1* Is Required for Full Virulence and Regulates Expression of Protein Effector and Secondary Metabolite Biosynthetic Genes. Mol. Plant-Microbe Interact..

[68] Arias S.L., Mary V.S., Otaiza S.N., Wunderlin D.A., Rubinstein H.R., Theumer M.G. (2016). Toxin Distribution and Sphingoid Base Imbalances in *Fusarium verticillioides*-Infected and Fumonisin B1-Watered Maize Seedlings. Phytochemistry.

[69] Otaiza-González S.N., Mary V.S., Arias S.L., Bertrand L., Velez P.A., Rodriguez M.G., Rubinstein H.R., Theumer M.G. (2022). Cell Death Induced by Fumonisin B1 in Two Maize Hybrids: Correlation with Oxidative Status Biomarkers and Salicylic and Jasmonic Acids Imbalances. Eur. J. Plant Pathol..

[70] Visentin I., Montis V., Döll K., Alabouvette C., Tamietti G., Karlovsky P., Cardinale F. (2012). Transcription of Genes in the Biosynthetic Pathway for Fumonisin Mycotoxins Is Epigenetically and Differentially Regulated in the Fungal Maize Pathogen *Fusarium verticillioides*. Eukaryot. Cell.

[71] Picot A., Barreau C., Pinson-Gadais L., Caron D., Lannou C., Richard-Forget F. (2010). Factors of the *Fusarium verticillioides* -Maize Environment Modulating Fumonisin Production. Crit. Rev. Microbiol..

[72] Flaherty J.E., Pirttilä A.M., Bluhm B.H., Woloshuk C.P. (2003). *PAC1*, a pH-Regulatory Gene from *Fusarium verticillioides*. Appl. Environ. Microbiol..

[73] Wiemann P., Willmann A., Straeten M., Kleigrewe K., Beyer M., Humpf H., Tudzynski B. (2009). Biosynthesis of the Red Pigment Bikaverin in *Fusarium fujikuroi*: Genes, Their Function and Regulation. Mol. Microbiol..

[74] Frandsen R.J.N., Rasmussen S.A., Knudsen P.B., Uhlig S., Petersen D., Lysøe E., Gotfredsen C.H., Giese H., Larsen T.O. (2016). Black Perithecial Pigmentation in *Fusarium* Species Is Due to the Accumulation of 5-Deoxybostrycoidin-Based Melanin. Sci. Rep..

[75] Proctor R.H., Butchko R.A.E., Brown D.W., Moretti A. (2007). Functional Characterization, Sequence Comparisons and Distribution of a Polyketide Synthase Gene Required for Perithecial Pigmentation in Some *Fusarium* Species. Food Addit. Contam..

[76] Zhang Y., Yu W., Lu Y., Wu Y., Ouyang Z., Tu Y., He B. (2024). Epigenetic Regulation of Fungal Secondary Metabolism. J. Fungi.

[77] Lamka G.F., Harder A.M., Sundaram M., Schwartz T.S., Christie M.R., DeWoody J.A., Willoughby J.R. (2022). Epigenetics in Ecology, Evolution, and Conservation. Front. Ecol. Evol..

[78] Herman J.J., Sultan S.E., Horgan-Kobelski T., Riggs C. (2012). Adaptive Transgenerational Plasticity in an Annual Plant: Grandparental and Parental Drought Stress Enhance Performance of Seedlings in Dry Soil. Integr. Comp. Biol..

[79] Sakashita A., Ooga M., Otsuka K., Maezawa S., Takeuchi C., Wakayama S., Wakayama T., Namekawa S.H. (2023). Polycomb Protein SCML2 Mediates Paternal Epigenetic Inheritance through Sperm Chromatin. Nucleic Acids Res..

[80] Reardon R.M., Walsh A.K., Larsen C.I., Schmidberger L.H., Morrow L.A., Thompson A.E., Wellik I.M., Thompson J.S. (2022). An Epigenetically Inherited UV Hyper-Resistance Phenotype in Saccharomyces Cerevisiae. Epigenetics Chromatin.

