# Supplementary material for: The Growth, Pathogenesis, and Secondary Metabolism of Fusarium verticillioides Are Epigenetically Modulated by Putative Heterochromatin Protein 1 (FvHP1)
Source: J Fungi (Basel). 2025 May 31;11(6):424. doi: 10.3390/jof11060424 (PMC12194461; doi:10.3390/jof11060424)
Supplement: Supplementary file 1 [file jof-11-00424-s001.zip › jof-3645148-supplementary.pdf]

Journal of *Fungi*

MDPI

Article

Supplementary material: The growth, pathogenesis, and secondary metabolism of *Fusarium verticillioides* are epigenetically modulated by putative heterochromatin protein 1 (FvHP1)

Andrés G. Jacquat [1,2], Natalia S. Podio [3], M. Carmen Cañizares [4], Pilar A. Velez [5,6], Martín G. Theumer [5,6], Vanessa A. Areco [7], M. Dolores Garcia-Pedrajas [4,*], José S. Dambolena [1,2,*]

[1] Facultad de Ciencias Exactas Físicas y Naturales (FCEFyN), Universidad Nacional de Córdoba (UNC), Córdoba 5000, Argentina; andresgjacquat@gmail.com (A.G.J), jdambolena@imbiv.unc.edu.ar (J.S.D.)
[2] Instituto Multidisciplinario de Biología Vegetal (IMBIV), Consejo Nacional de Investigaciones Científicas y Técnicas (CONICET), Avenida Vélez Sarsfield 1611, Córdoba X5016GCA, Argentina
[3] Instituto de Ciencia y Tecnología de Alimentos Córdoba (ICYTAC), CONICET and Facultad de Ciencias Químicas (FCQ), Universidad Nacional de Córdoba, Bv. Dr. Juan Filloy s/n; Cdad. Universitaria, 5000, Córdoba, Argentina; npodio@fcq.unc.edu.ar
[4] Instituto de Hortofruticultura Subtropical y Mediterránea "La Mayora", Universidad de Málaga, Consejo Superior de Investigaciones Científicas (IHSM—UMA—CSIC), Estación Experimental "La Mayora", Avenida Dr. Wienberg s/n, Algarrobo-Costa 29750, Málaga, Spain; mariola@eelm.csic.es (M. D. G. P); carmen.canizares@eelm.csic.es (M. C. C.)
[5] Departamento de Bioquímica Clínica, Facultad de Ciencias Químicas (FCQ), Universidad Nacional de Córdoba (UNC), Córdoba 5000, Argentina; mgtheumer@fcq.unc.edu.ar (M. G. T.), pilarvelez@fcq.unc.edu.a (P. A. V.)
[6] Centro de Investigaciones en Bioquímica Clínica e Inmunología (CIBICI), Consejo Nacional de Investigaciones Científicas y Técnicas (CONICET), Haya de la Torre y Medina Allende - Ciudad Universitaria - Córdoba X5000HUA, Argentina
[7] Instituto Multidisciplinario de Investigación y Transferencia Agroalimentaria y Biotecnológica (IMITAB, CONICET-UNVM); vareco@unvm.edu.ar

* Correspondence: mariola@eelm.csic.es (M.D.G.P); jdambolena@imbiv.unc.edu.ar (J.S.D.)

Academic Editor: Firstname Lastname

Received: date
Revised: date
Accepted: date
Published: date

Citation: To be added by editorial staff during production.

Copyright: © 2025 by the authors. Submitted for possible open access publication under the terms and conditions of the Creative Commons Attribution (CC BY) license (https://creativecommons.org/licenses/by/4.0/).

Abstract: *Fusarium verticillioides* is a globally prevalent phytopathogenic fungus responsible for multiple diseases in maize and is a major producer of the mycotoxin fumonisin B1 (FB1) a highly toxic fungal secondary metabolite (FSM). The histone code, which includes reversible modifications such as acetylation and methylation, plays a critical role in regulating chromatin structure and gene expression. In fungi, di- and tri-methylation of histone H3 at lysine 9 (H3K9me2/3) serves as a key epigenetic mark associated with heterochromatin formation and transcriptional repression. In this study, we identified and characterized a putative heterochromatin protein 1 (HP1) family member in *F. verticillioides*, designated FvHP1, based on conserved domain architecture and phylogenetic analyses. FvHP1 retains essential residues required for H3K9me2/3 recognition, supporting its functional conservation within the HP1 protein family. Phenotypic analysis of the ΔFvHP1 mutant revealed impaired vegetative growth, reduced conidiation and virulence, and altered FB1 mycotoxin production. Additionally, the accumulation of red pigment in the mutant was linked to the deregulation of secondary metabolism, specifically the overproduction of fusarubin-type naphthoquinones, such as 8-O-methylnectriafurone

(FSM). These results support the role of FvHP1 in facultative heterochromatin-mediated repression of sub-telomeric biosynthetic gene clusters, including the pigment-associated PGL1 cluster. Our findings provide new insights into the epigenetic regulation of fungal pathogenicity and metabolite production, as well as the first evidence of a functional HP1 homolog in *F. verticillioides*.

Keywords: *Fusarium verticillioides*; phytopathogenic; fumonisin B1; fungal secondary metabolite; histone code, epigenetic, heterochromatin, heterochromatin protein 1 (HP1), fusarubin-type naphthoquinones; 8-O-methylnectriafurone

Supplementary material

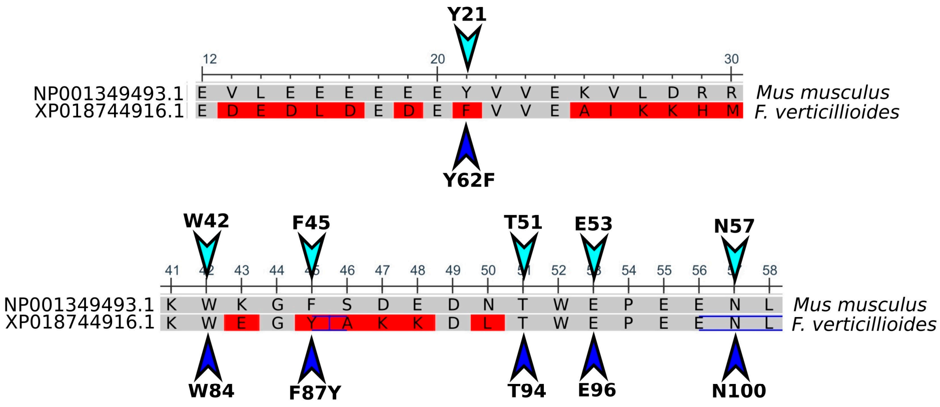

Figure S1. Chromo domain sequences pair alignment (NCBI MSA Viewer v1.20.1) of FvHP1 (this study) and mouse HP1β (also known as MOD1 or M31). Arrows indicate the key sites for the recognition of 2-/3-methyl lysine 9 of H3 histone according to **Nielsen et al. (2002)** (see main text). Note that the residues at positions 62 and 87 of FvHP1 have undergone a mutation, resulting in a substitution with another residue of similar chemical nature. The substitution particularly occurred between a residue of F and Y. According to the analysis by **Nielsen et al. (2002)**, this type of substitution is common among members of the HP1 family and apparently does not affect their functionality.

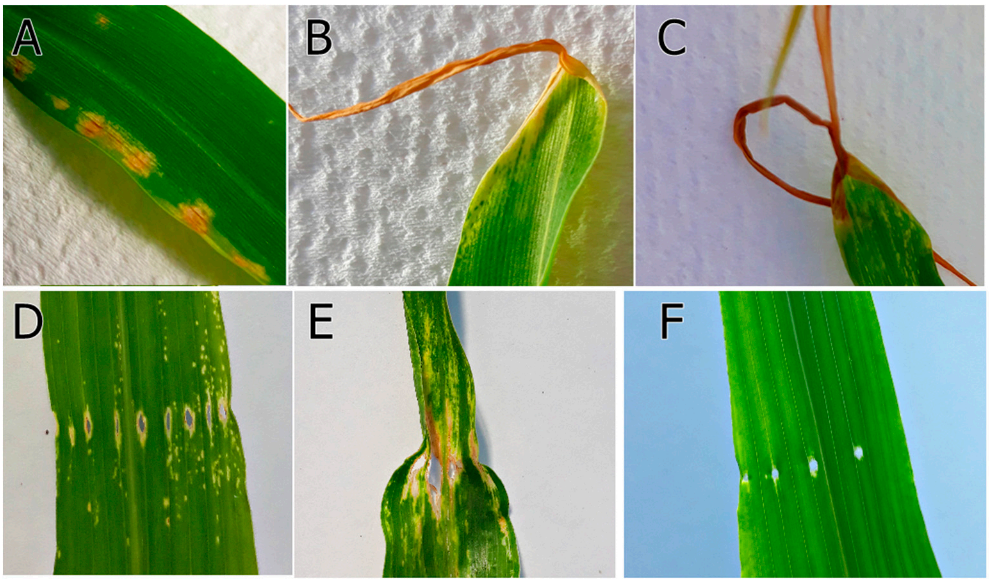

Figure S2. Signs of necrosis on a maize seedling leaf. A) irregular spots along the leaf blade. B) Necrosis and wilting at the tip of the leaf blade. C) Necrosis and wilting with ends "attached" to other leaves. D) irregular necrotic spots are "emerging" from the injection site lesion. E) Necrotic spots with tissue atrophy at the injection site lesion. For comparison, a photo of the injection site lesion of a control seedling (inoculated with sterile water) is shown. All photographs were taken during the greenhouse pathogenicity assay. A Samsung SM-J530F digital camera was used to capture images, which were then edited using PhotoScape V3.7.

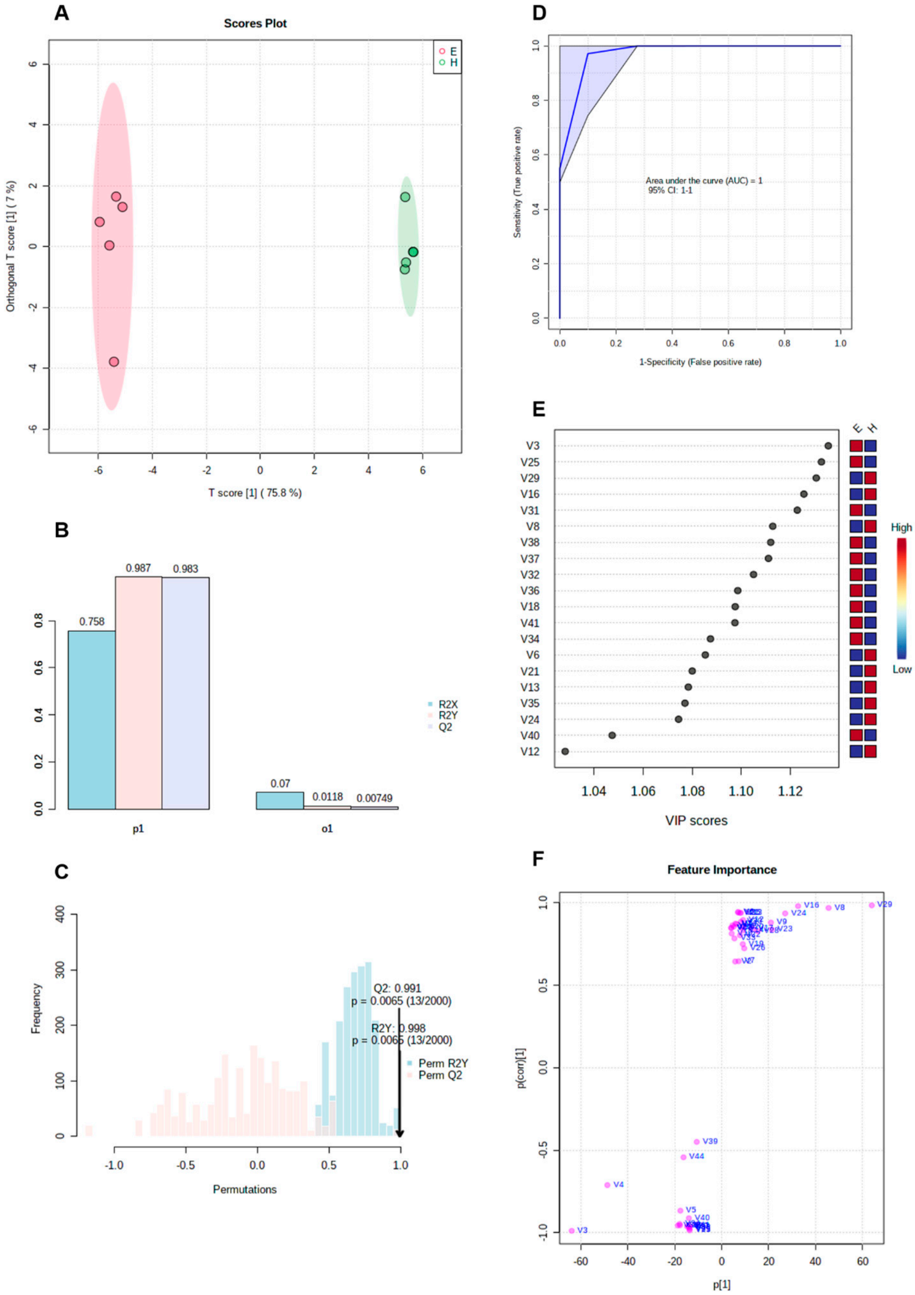

Figure S3. Orthogonal Partial Least Squares Discriminant Analysis (OPLS-DA) to discriminate metabolite fungal strain. Samples normalized by sum and Pareto scaled. (A) Scores plot, (B) Correlation and predictive parameters (C) Permutation test, (D) VIP values of the OPLS-DA model, and (E) S-plots. Feature Importance (F).

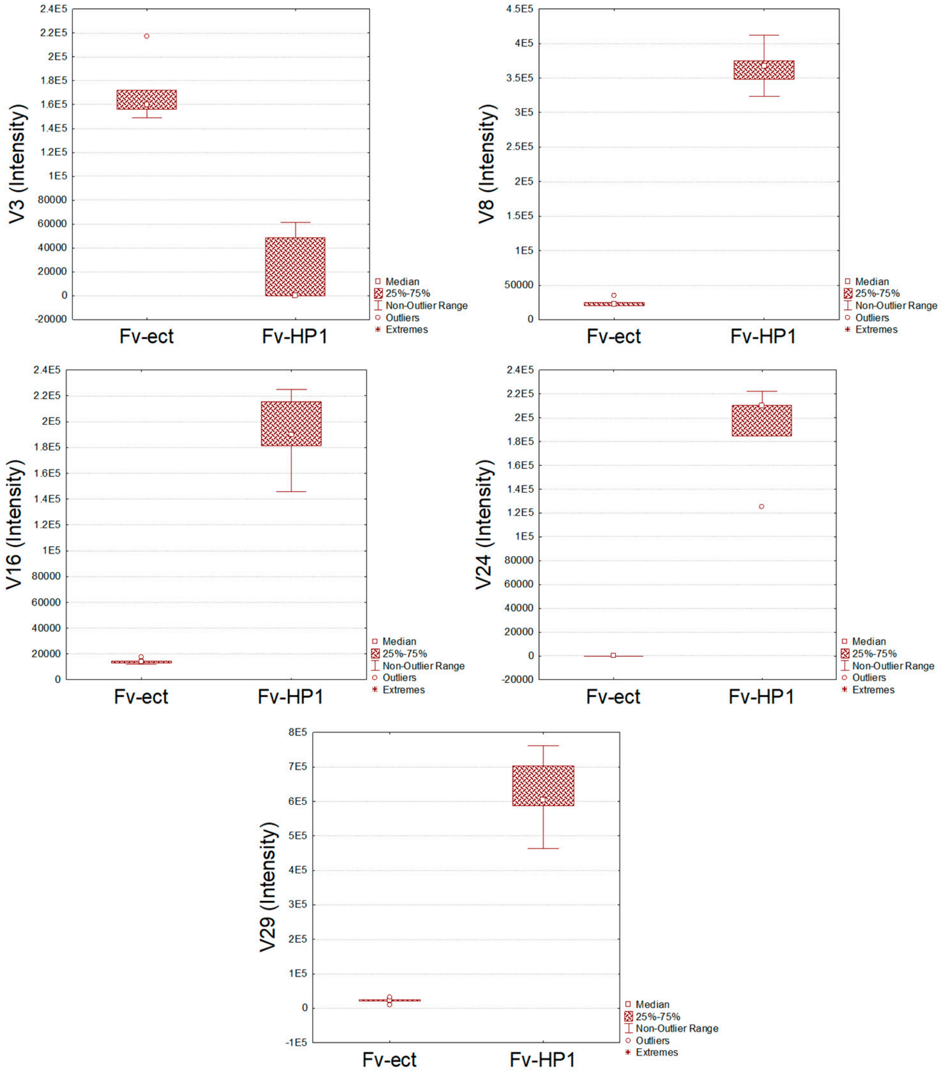

Figure S4. Box Plot of the most important metabolites to discriminate between ΔFvHP1 and FvECT strains.

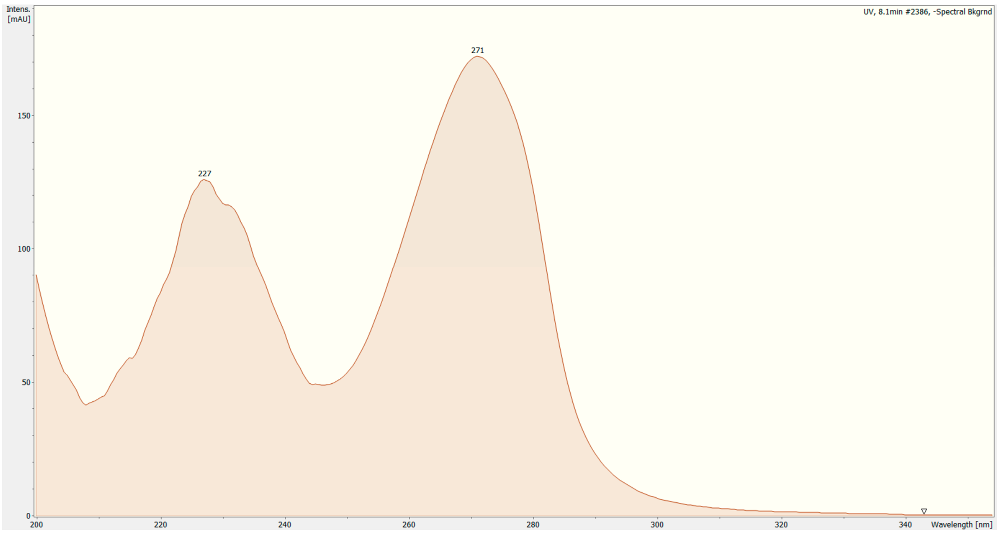

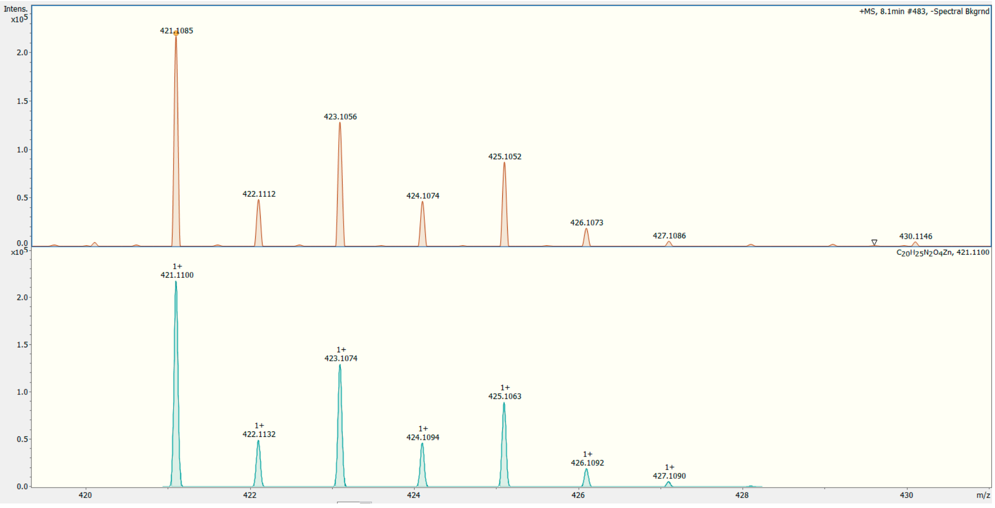

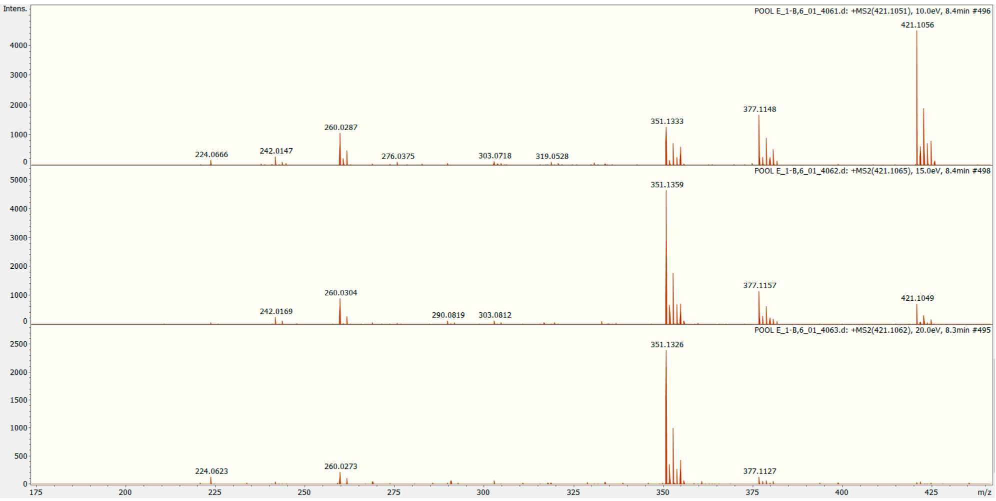

Figure S5. UV-Vis, MS, and MSMS spectrums of Metabolite V3.

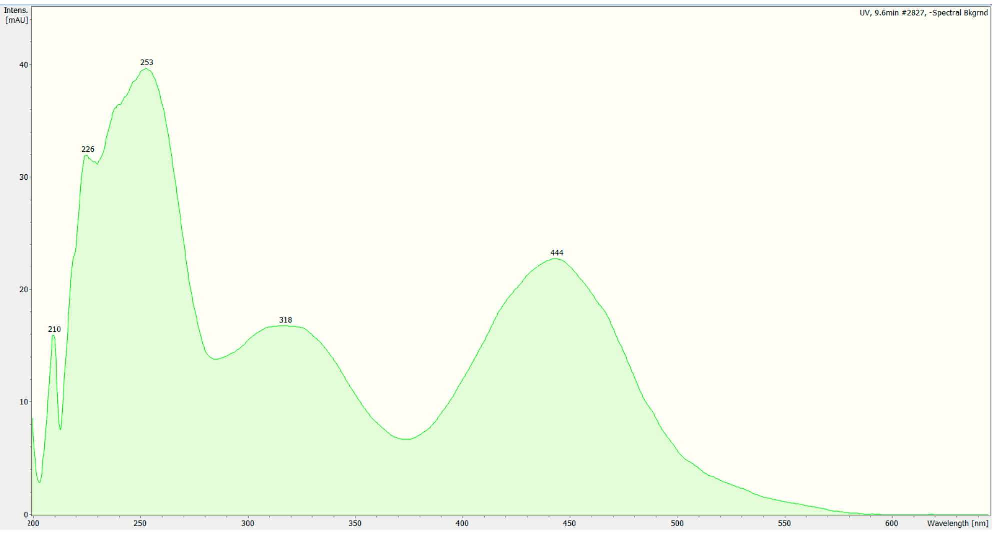

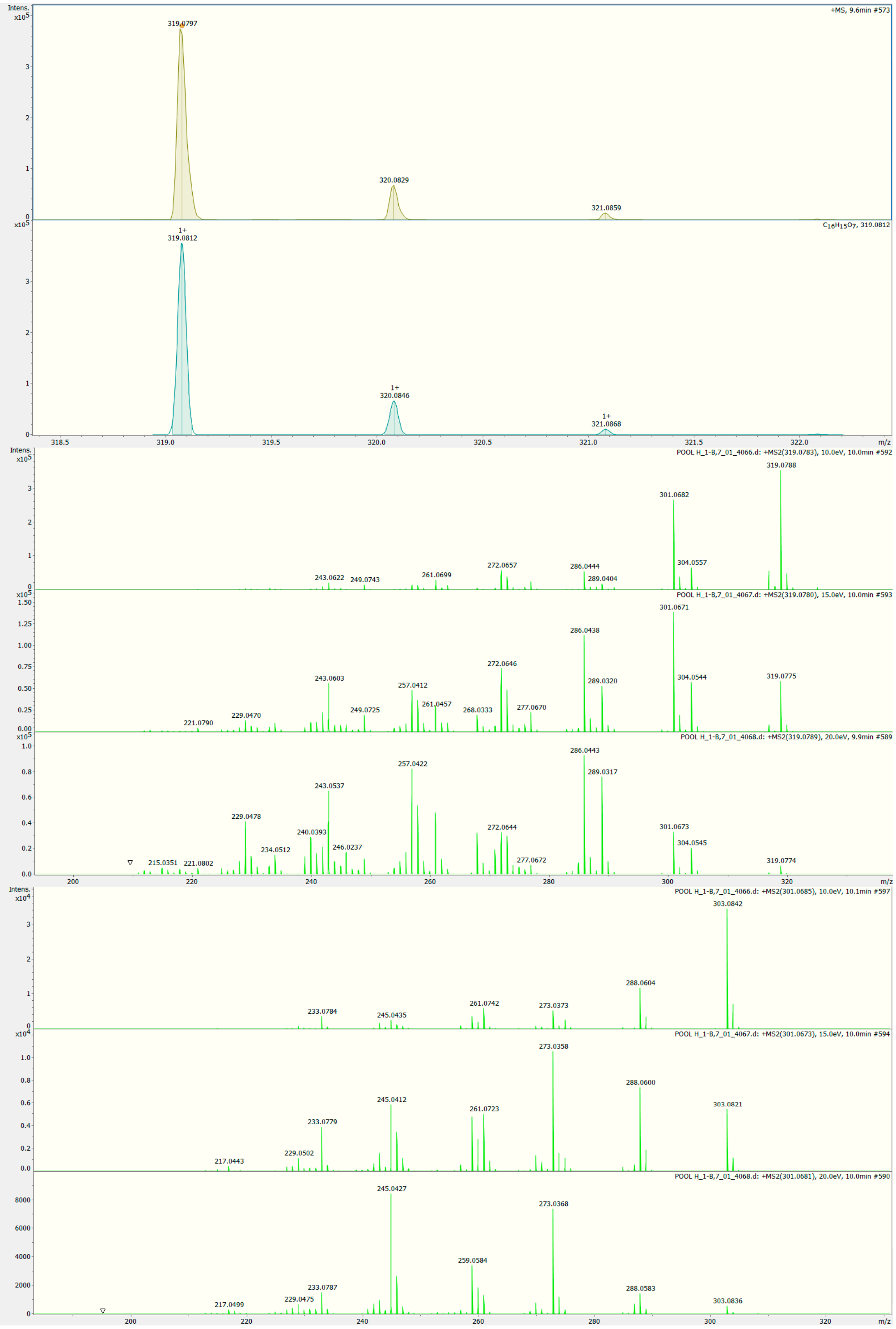
Intens.
x10^5
+MS, 9.6min #573
319.0797
320.0829
321.0859
C16H15O7, 319.0812
1+
319.0812
1+
320.0846
1+
321.0868
318.5
319.0
319.5
320.0
320.5
321.0
321.5
322.0
m/z
POOL H_1-B,7_01_4066.d: +MS2(319.0783), 10.0eV, 10.0min #592
319.0788
301.0682
304.0557
286.0444
289.0404
272.0657
261.0699
243.0622
249.0743
POOL H_1-B,7_01_4067.d: +MS2(319.0780), 15.0eV, 10.0min #593
301.0671
286.0438
272.0646
243.0603
289.0320
304.0544
319.0775
257.0412
261.0457
268.0333
277.0670
249.0725
229.0470
221.0790
POOL H_1-B,7_01_4068.d: +MS2(319.0789), 20.0eV, 9.9min #589
286.0443
257.0422
289.0317
243.0537
229.0478
240.0393
272.0644
301.0673
304.0545
234.0512
246.0237
277.0672
319.0774
215.0351
221.0802
200
220
240
260
280
300
320
m/z
POOL H_1-B,7_01_4066.d: +MS2(301.0685), 10.0eV, 10.1min #597
303.0842
288.0604
261.0742
273.0373
233.0784
245.0435
POOL H_1-B,7_01_4067.d: +MS2(301.0673), 15.0eV, 10.0min #594
273.0358
288.0600
245.0412
261.0723
303.0821
233.0779
229.0502
217.0443
POOL H_1-B,7_01_4068.d: +MS2(301.0681), 20.0eV, 10.0min #590
245.0427
273.0368
259.0584
233.0787
229.0475
217.0499
288.0583
303.0836
200
220
240
260
280
300
320
m/z

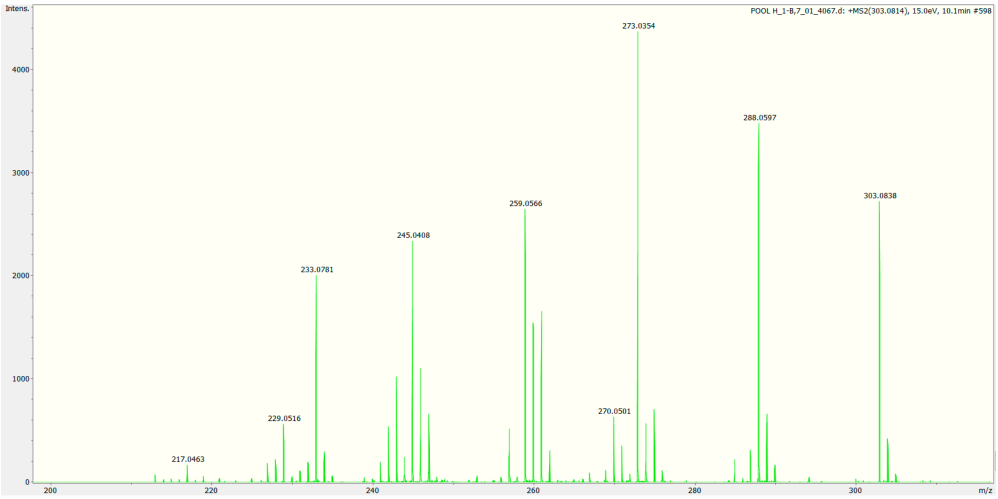

Figure S6. UV-Vis, MS and MSMS spectrums of Metabolite V8. MS² spectrums were also included.

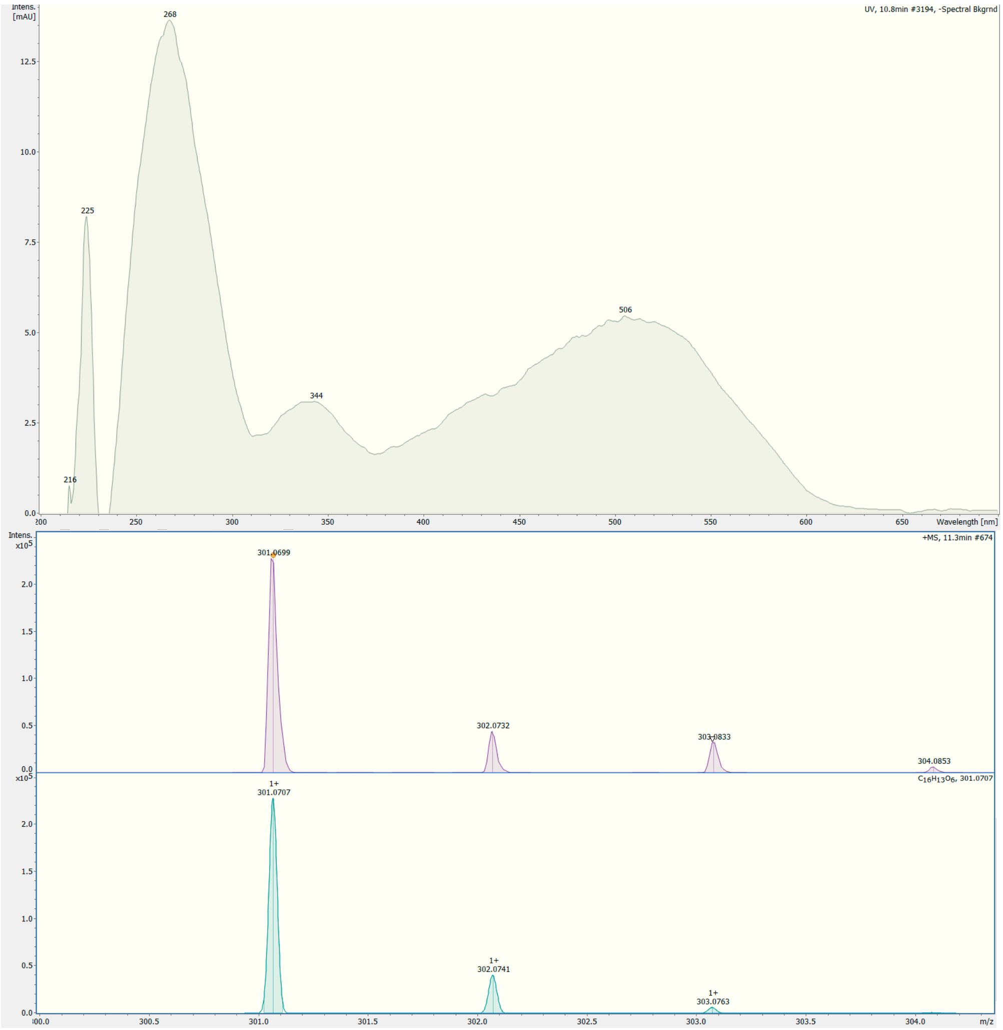

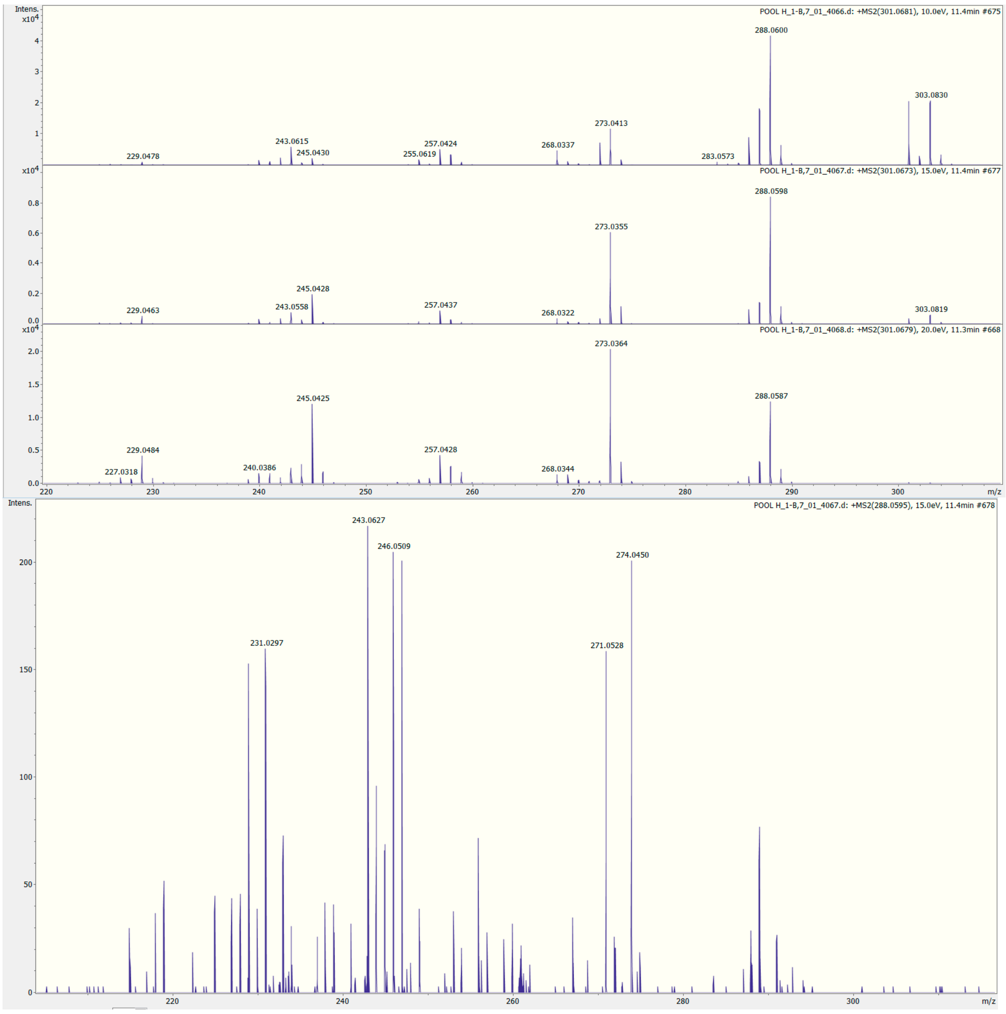

Figure S7. UV-Vis, MS, and MSMS spectrums of Metabolite V16.

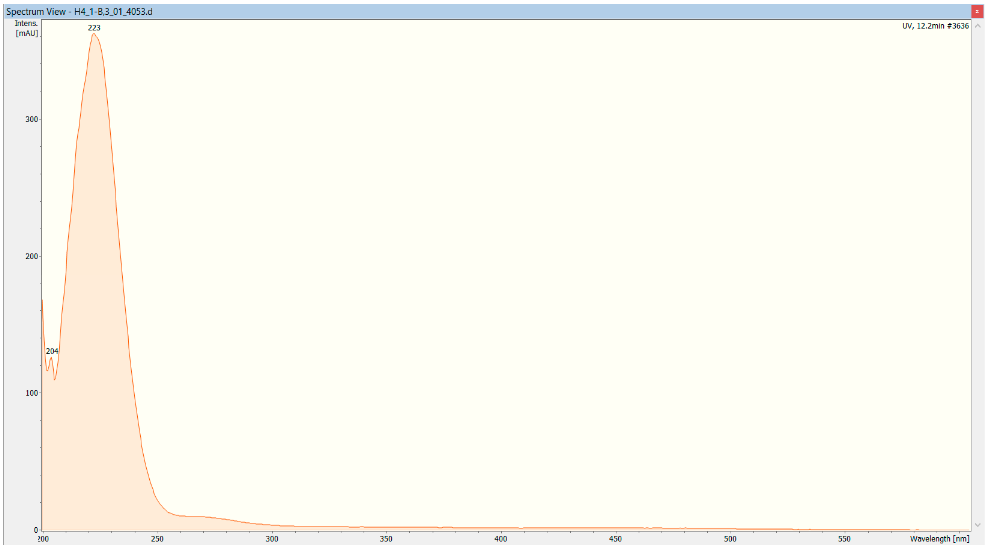

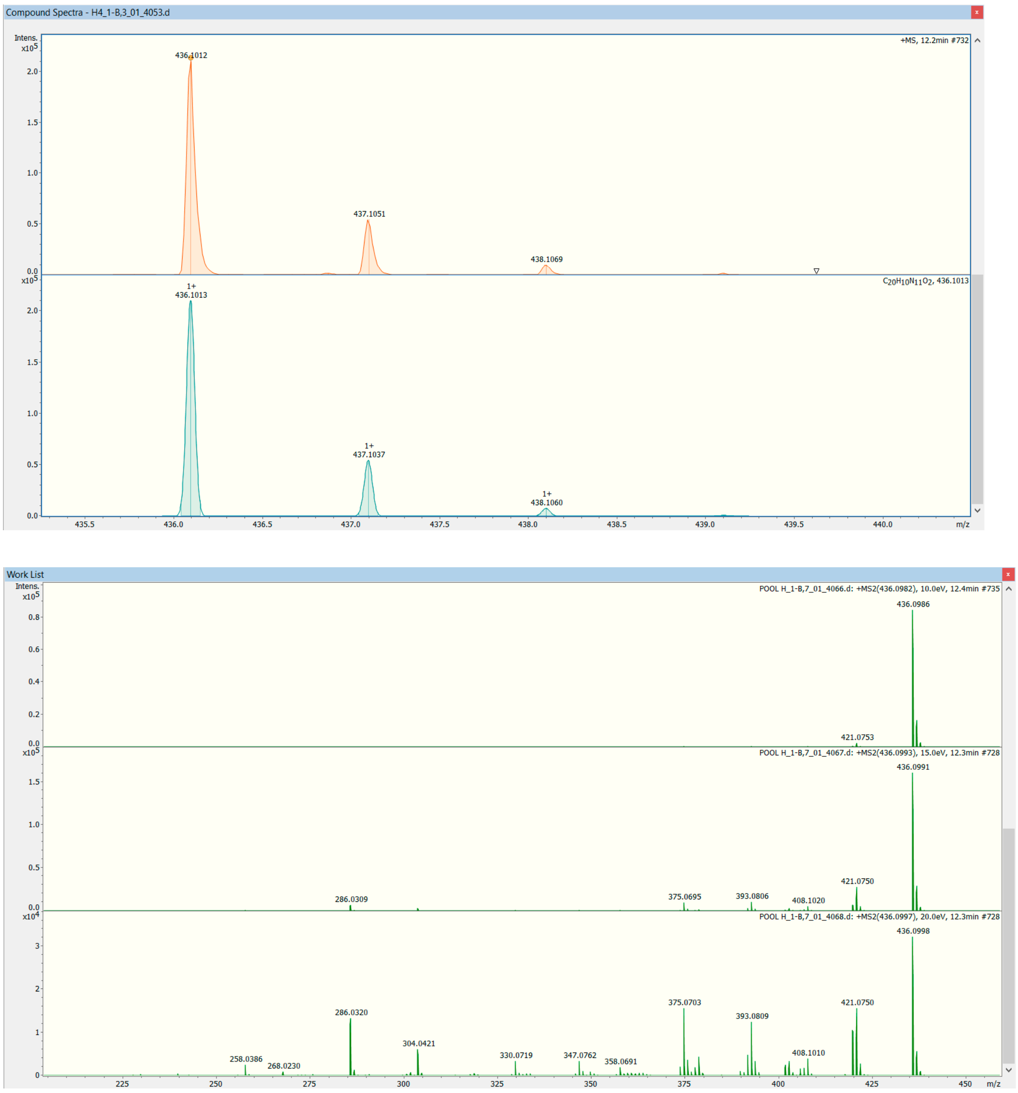

Figure S8. UV-Vis, MS, and MSMS spectrums of Metabolite V24.

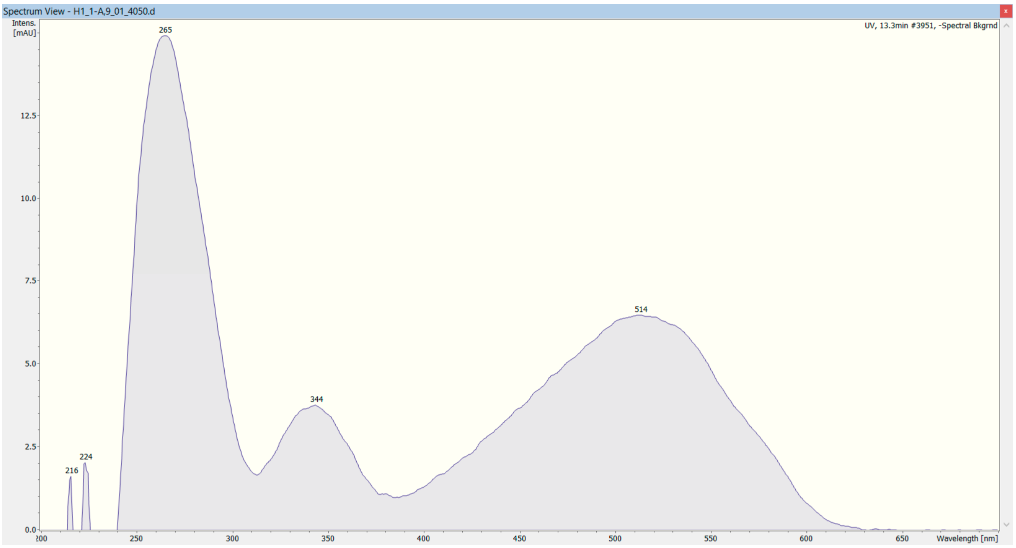

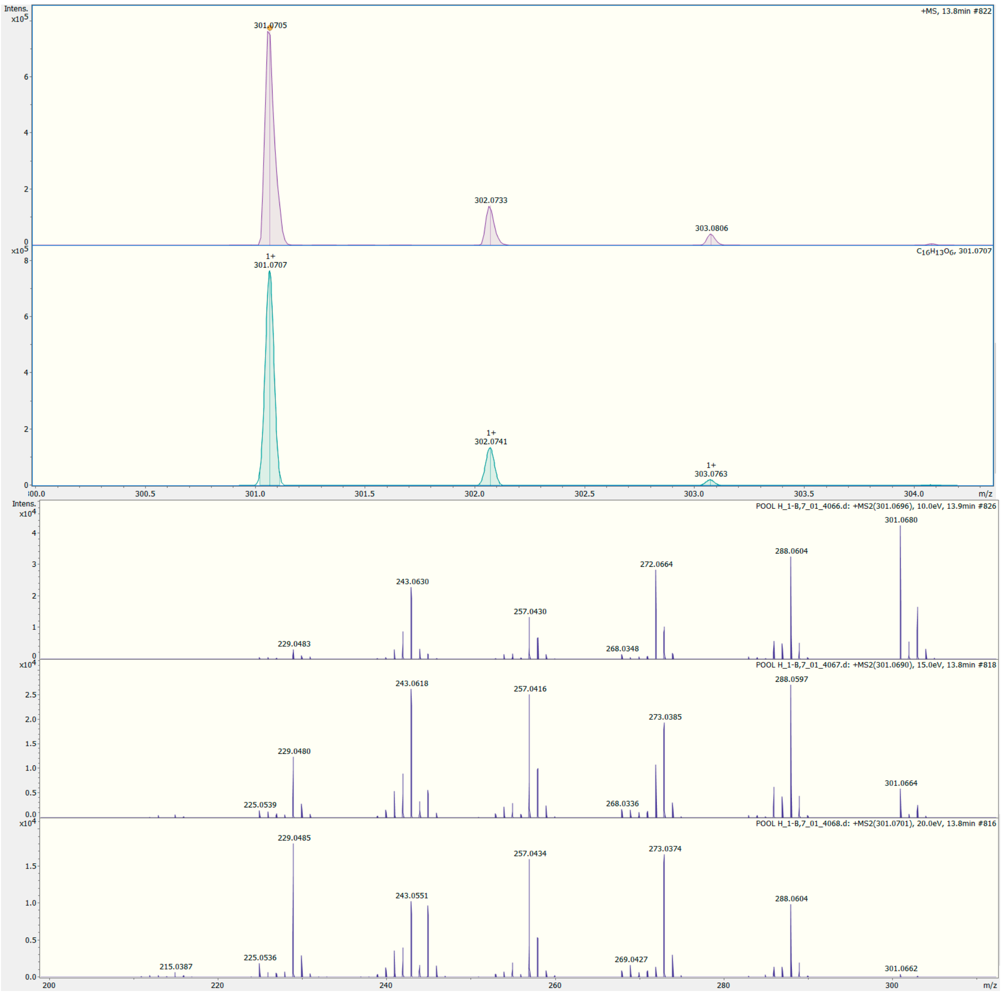

Figure S9. UV-Vis, MS, and MSMS spectrums of Metabolite V29.
